# Sex and sex hormonal regulation of the atrial inward rectifier potassium current (I_K1_): insights into potential pro-arrhythmic mechanisms

**DOI:** 10.1093/cvr/cvaf074

**Published:** 2025-04-24

**Authors:** Lucilla Giammarino, Lluis Matas, Nicolò Alerni, András Horváth, Varjany Vashanthakumar, Saranda Nimani, Miriam Barbieri, Sahej Bains, Ruben Lopez, Julien Louradour, Balazs Ördög, Thomas Hof, Ange Maguy, Giulio Conte, Angelo Auricchio, Ulrich Schotten, Katja E Odening

**Affiliations:** Translational Cardiology, Department of Physiology and Department of Cardiology, University of Bern, University Hospital Bern, Bühlplatz 5, Bern 3012, Switzerland; Translational Cardiology, Department of Physiology and Department of Cardiology, University of Bern, University Hospital Bern, Bühlplatz 5, Bern 3012, Switzerland; Translational Cardiology, Department of Physiology and Department of Cardiology, University of Bern, University Hospital Bern, Bühlplatz 5, Bern 3012, Switzerland; Translational Cardiology, Department of Physiology and Department of Cardiology, University of Bern, University Hospital Bern, Bühlplatz 5, Bern 3012, Switzerland; Translational Cardiology, Department of Physiology and Department of Cardiology, University of Bern, University Hospital Bern, Bühlplatz 5, Bern 3012, Switzerland; Translational Cardiology, Department of Physiology and Department of Cardiology, University of Bern, University Hospital Bern, Bühlplatz 5, Bern 3012, Switzerland; Translational Cardiology, Department of Physiology and Department of Cardiology, University of Bern, University Hospital Bern, Bühlplatz 5, Bern 3012, Switzerland; Departments of Cardiovascular Medicine, Pediatric and Adolescent Medicine, and Molecular Pharmacology and Experimental Therapeutics, Divisions of Heart Rhythm Services and Pediatric Cardiology, Windland Smith Rice Genetic Heart Rhythm Clinic and the Windland Smith Rice Sudden Death Genomics Laboratory, Mayo Clinic, Rochester, USA; Translational Cardiology, Department of Physiology and Department of Cardiology, University of Bern, University Hospital Bern, Bühlplatz 5, Bern 3012, Switzerland; Translational Cardiology, Department of Physiology and Department of Cardiology, University of Bern, University Hospital Bern, Bühlplatz 5, Bern 3012, Switzerland; Translational Cardiology, Department of Physiology and Department of Cardiology, University of Bern, University Hospital Bern, Bühlplatz 5, Bern 3012, Switzerland; Translational Cardiology, Department of Physiology and Department of Cardiology, University of Bern, University Hospital Bern, Bühlplatz 5, Bern 3012, Switzerland; Department of Physiology, University of Bern, Bern, Switzerland; Department of Cardiology, Cardiocentro Ticino Institute, Ente Ospedaliero Cantonale, Lugano, Switzerland; Biomedical Sciences, Università della Svizzera Italiana, Lugano, Switzerland; Department of Cardiology, Cardiocentro Ticino Institute, Ente Ospedaliero Cantonale, Lugano, Switzerland; Biomedical Sciences, Università della Svizzera Italiana, Lugano, Switzerland; Department of Physiology, Maastricht University, Maastricht, The Netherlands; Department of Cardiology, Maastricht University Medical Centre+, Maastricht, The Netherlands; Translational Cardiology, Department of Physiology and Department of Cardiology, University of Bern, University Hospital Bern, Bühlplatz 5, Bern 3012, Switzerland

**Keywords:** Atrial fibrillation, Sex differences, Sex hormones, I_K1_

## Abstract

**Aims:**

Pronounced sex-differences are known in the incidence of atrial fibrillation (AF). In this study, we aimed to investigate the atrial electrophysiological properties that may underlie sex-differences in AF incidence in the younger population, focusing on I_K1_, a cardiac ion current important for action potential (AP) stability and triggered activity.

**Methods and results:**

We assessed sex-differences in P-wave morphology in 12-lead ECG in healthy young New Zealand White rabbits. Males presented longer PWD and larger P-wave area compared to females. Patch-clamp experiments were performed in isolated rabbit atrial cardiomyocytes (CMs). Male atrial CMs presented higher delayed after depolarizations (DAD) incidence, amplitude, and area under the curve (AUC) than females, potentially facilitating the presence of atrial triggered activity in males. Male atrial CMs showed a less hyperpolarized resting membrane potential (RMP), a 50% smaller I_K1_, and a 26% reduction in K_ir2.1_ protein expression, a pore forming subunit of I_K1_, than females. Dihydrotestosterone (DHT) effects were investigated acutely and semi-chronically *ex vivo*. Experiments showed that the sex-difference in I_K1_ could be mimicked by DHT. In female atrial CMs, acute and semi-chronic (24 h) DHT administration reduced I_K1_. In the presence of a PKC-inhibitor, DHT-mediated I_K1_ reduction was not observed in atrial female CMs, suggesting it to be PKC-mediated. Chronic DHT-effects were investigated *in vivo* in female rabbits after hormone-releasing pellet implantation. After 2 weeks, animals showed a significantly prolonged and larger P-wave, a smaller atrial I_K1_ and a trend towards an increased DAD amplitude and AUC.

**Conclusion:**

Sex impacts on atrial electrophysiology, leading to sex-differences in P-wave morphology, triggered activity, RMP, and I_K1_. These sex-differences can be mimicked by sex hormone-treatment, suggesting that sex hormones—particularly DHT—play a pivotal role in mediating sex-differences in atrial electrophysiology. Such sex-differences might impact on the propensity to develop AF, particularly in the younger population.

## Graphical Abstract

**Graphical Abstract:**
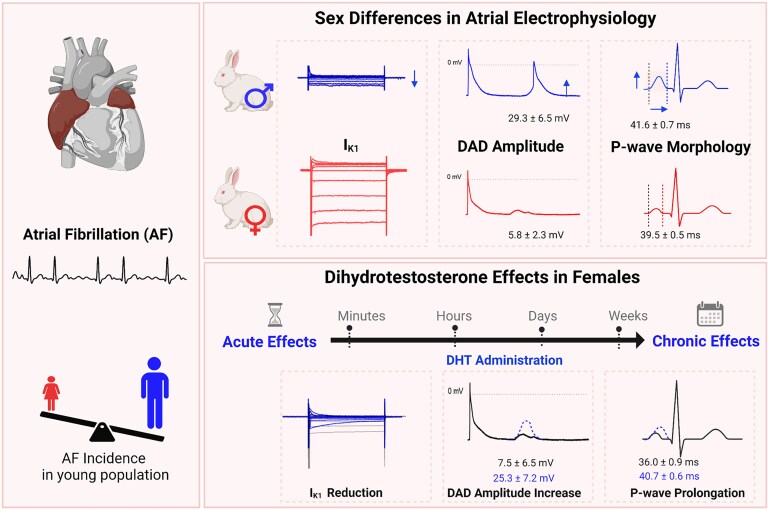

**Time of primary review: 27 days**

**See the editorial comment for this article ‘Testosterone and atrial fibrillation: does the dose make the poison?’, by F. E. Mason *et al*., https://doi.org/10.1093/cvr/cvaf113.**

## 1. Introduction

Atrial fibrillation (AF) is the most common cardiac arrhythmia, markedly affecting quality of life, morbidity, and mortality. Age and sex are the two most powerful predictors of AF incidence. Although the prevalence of AF doubles with each decade of age, male sex is associated with a greater risk of developing AF even after adjusting for age and pre-disposing conditions.^1–3^

Differences between men and women in AF incidence, recurrence rates, risk factors, comorbidities, underlying mechanisms, and response to treatment have been largely reported in the literature.^4,5^ Overall, men carry a higher risk for AF development than women. Notably, at younger, pre-menopausal age, women appear to possess protecting mechanisms against AF.^5^ A plausible explanation for these observed sex differences in AF in the young population could be the impact of sex hormones. Different clinical studies have shed light on the effects of sex hormones, demonstrating a correlation between hormonal replacement therapies in women and a reduced AF incidence.^6,7^ For example, women undergoing anti-oestrogen treatment present an increased AF risk, hinting towards a potential protective role of estrogen.^8^ In contrast, a population-based longitudinal study demonstrated that higher testosterone levels were associated with a higher incidence of AF in men,^9^ suggesting potential pro-arrhythmic effects of testosterone. Importantly, recent studies reported a biphasic association of total testosterone level with incident AF risk: while a 1 nmol/L increase in total testosterone (in healthy men) was associated with a 18% higher risk of AF,^10^ in a study in men with initially low testosterone levels, a normalization of testosterone levels through hormone replacement therapy led to a significant decrease in the incidence of AF.^11^ These observations indicate that dependent on the initial testosterone level, an increase in testosterone may have pro- or anti-arrhythmic effects. The mechanisms behind the impact of sex hormones on AF incidence, however, are thus far unknown.

Several parameters defining sex-differences in cardiac electrophysiology (ECG) may potentially affect the risk for arrhythmias. A promising candidate in modulating sex differences in atrial arrhythmias is the inward rectifier potassium current (I_K1_). I_K1_ is responsible for shaping the final repolarization phase of the action potential (AP) as well as for stabilizing the resting membrane potential (RMP) below the threshold for spontaneous depolarizations.^12^ Recent studies have described the role of I_K1_ in ventricular arrhythmias and suggested a possible role also in supraventricular arrhythmias.^12–14^ A decrease in I_K1_ may facilitate the presence of afterdepolarizations, while an increase may facilitate re-entrant arrhythmias.^12^ Therefore, potential sex-hormone effects on atrial I_K1_ would likely have a profound effect on cardiac excitability and may contribute to sex-specific differences in atrial arrhythmias incidence.

Based on the observed pronounced sex differences in AF incidence in the young population (in which confounding comorbidities are largely missing), we hypothesized that testosterone could exert pro-arrhythmic and/or estradiol could exert anti-arrhythmic, protective effects. In this study, we therefore investigated sex differences in atrial electrical properties and the impact of sex hormones on atrial I_K1_ for a better understanding of sex-related supraventricular arrhythmia mechanisms in a healthy young population of rabbits, a particularly suitable species for research in ECG. Indeed, rabbits show pronounced similarities with humans in terms of ECG, repolarizing potassium channels (I_Ks_, I_Kr_, and I_K1_) and AP characteristics,^15^ facilitating translation of mechanistic findings to human (patho)physiology. Furthermore, using rabbits allowed us to investigate both, acute and more chronic effects of sex hormones on the cellular level and *in vivo*.

## 2. Methods

### 2.1 Animal experiments

All animal experiments were performed in compliance with EU legislation (directive 2010/63/EU) and the Swiss Animal Welfare Ordinance, after approval by the Cantonal Veterinary Office and the Animal Welfare Officer (Kanton Bern, approval number BE131-20). Adult New Zealand White rabbits of both sexes (4–6 months old, 3–5 kg) from in-house breeding colonies or purchased from a commercial provider, Charles River Laboratories (CRL, France), were used for experiments. Female breeders in our in-house breeding facility originate from the same CRL facility as the few animals purchased from commercial breeding, ensuring a fairly similar genetic background. In addition, prior to experiments, we performed comparative tests on P-wave morphology and I_K1_ current density between the in-house bred and commercially sourced rabbits to ensure that the rabbits were comparable. Animal housing and handling was in accordance with good animal practice as defined by the Federation of European Laboratory Animal Science Association, FELASA.

### 2.2 Conventional 12-lead surface ECG

For *in vivo* experiments, rabbits were anesthetized with an intra-muscular injection of ketamine S (Ketanest S®, Pfizer) and xylazine (Rompun® 2%, Bayer) (12.5 mg/kg, 3.75 mg/kg respectively), which have been shown not to alter ECG parameters in previous studies.^16^ 12-Lead surface ECG recordings were performed, using an ECG machine (Custo med, Germany). Similarly, 12-lead ECG on awake, healthy, young volunteers, without history of AF were performed after written consent and approval by the local ethics committee in compliance with the declaration of Helsinki. ECG signal analysis was performed manually by considering 10 consecutive beats in lead II, using a software (Custo diagnostic, Custo med, Germany) to allow precise measurement.

### 2.3 Isolation and culture of rabbit atrial cardiomyocytes

Rabbit atrial cardiomyocytes (CMs) were isolated in Langendorff-perfused hearts from both, left (LA) and right atria (RA), in all animals using an enzymatic digestion. After ketamine S/xylazine anaesthesia and euthanasia via pentobarbital intra-venous (i.v.) overdose injection (Esconarkon, 150 mg/kg), the heart was rapidly extracted. The heart was mounted on a Langendorff perfusion system (Hugo Sachs Elektronik—Harvard Apparatus GmbH, Germany) and perfused for 5 min with oxygenated, body-temperature, Tyrode solution containing (in mM): 135 NaCl, 0.4 NaH_2_PO_4_, 5 KCl, 10 HEPES, 1 MgCl_2_, 10 Glucose, 10 Creatine, 20 Taurine (pH = 7.6 using NaOH). Afterwards, the perfusion was switched to a 0.1 mM EGTA-supplemented Tyrode until the heart stopped contracting and followed by a collagenase (Worthington type 1, Worthington Biochemical, USA) digestion step, 200 mg in 80 µM Ca^2+^ Tyrode. The digestion was performed for around 20–25 min, until atrial tissue appeared almost transparent. Isolated CMs were stored in 0.2 mM Ca^2+^ Tyrode for immediate patch-clamp experiments or cultured in laminin-coated dishes (Sigma, USA) for 24 h with or without sex hormones to evaluate the semi-chronic effects on I_K1_. 17β-Estradiol (Sigma Chemical, USA) and dihydrotestosterone (DHT) (Selleckchem, USA) were dissolved in DMSO to a stock solution of 50 mM. Concentrations tested were 10 nM, 3 µM, and 10 µM. Culturing medium contained: 5% fetal bovine serum, 1% penicillin/streptomycin, 5 mM Creatinine, 2 mM L-carnitine, 5 mM Taurine, 1 mM Na-Pyruvate, 0.1 µM Insulin, 0.01 mM Cytosine-B-D-arabinofurnoside, and 5 µg/mL gentamycin in M199 medium.^17^

### 2.4 Patch-clamp experiments in isolated atrial CMs

Whole-cell patch-clamp technique was used to measure the membrane currents from isolated atrial CM using an Axopatch-200B patch-clamp amplifier and Axon Digidata 1550B (Molecular Devices, USA) controlled by pCLAMP software (Molecular Devices, USA), and a HEKA EPC Plus 10 amplifier with Patchmaster software (Multichannel system, Germany). These softwares were used to produce voltage pulse protocols, data acquisition, and analysis. All the recordings were carried out at 37°C, using ThermoClamp-1 temperature controller (Digitimer, UK). Borosilicate glass pipettes were pulled by a DMZ universal puller (Zeitz Instruments, Germany) with resistance of around 2–2.5 MΩ when filled with pipette internal solution. The chamber was continuously superfused with the external recording solution.

For I_K1_ recordings, pipette solution composition was (in mM): 125 KCl, 5 NaCl, 1 MgCl_2_, 5 K_2_ATP, 10 HEPES, 5 EGTA, and the extracellular solution consisted of Tyrode solution supplemented with 1.8 mM Ca^2+^. I_K1_ was elicited by voltage steps of 500 ms from −120 to −30 mV in 10 mV increment steps at 1 Hz frequency. Individual currents were expressed as densities (pA/pF) to normalize for changes in cell capacitance. I_K1_ was measured as the steady-state current at the end of the pulse; leak current was subtracted. Cells displaying more than 100 pA leak current were excluded. The holding potential in atrial CM was −80 mV. RMP of isolated atrial CMs was measured using current-clamp mode.

For AP recordings, pipette solution consisted of (mM): 125 KCl, 5 NaCl, 1 MgCl_2_, 5 K_2_ATP, 10 HEPES, and 0.5 EGTA. APs were recorded using 1.8 mM Ca^2+^ Tyrode extracellular solution at a stimulation rate of 1 Hz. A small amount (≤500 pA) of negative current was injected during the recordings to stabilize the atrial CMs. Several AP parameters were assessed, including RMP, action potential amplitude (APA), AP duration at 90% (APD_90_) of total repolarization, and maximum upstroke velocity of the AP (dV/dTmax) in a subset of atrial CMs without any (or very small ≤30 pA) current injection.

### 2.5 AP recordings in rabbit atrial tissues

Sharp-electrode technique was used to record AP in physiological, intact atrial tissue preparations. To record atrial APs, glass microelectrodes (GC200F-15, PHYMEP, France) were pulled using a DMZ universal puller (Zeitz Instruments, Germany) and filled with 3 M KCl (30 to 40 MΩ). After ketamine S/xylazine anaesthesia and euthanasia via pentobarbital i.v. injection, the heart was rapidly extracted. Left atrium and right atrium were collected and pinned down, exposing the endothelium, superfused at a rate of 20 mL/min with a physiological solution at 37°C, gassed with a 95% O_2_ and 5% CO_2_ mixture. The physiological bath solution used (pH 7.4) contained the following (in mM): NaCl 108, KCl 4, CaCl_2_ 1.8, MgCl_2_ 1, NaH_2_PO_4_ 1.8, NaH_2_CO_3_ 25, and glucose 11, and was equilibrated with O_2_–CO_2_ (95:5).

Atria were paced at 1 Hz with an external Isostim A320 point stimulator (World Precision Instruments, USA), connected to a bipolar silver-wire electrode. After 2 h, transmembrane potential and APs were recorded after cell impalement. Microelectrodes were coupled to the input stages of an impedance capacitance-neutralizing Electro 705 amplifier (World Precision Instruments, USA) and APs were displayed and analysed using an iox 2 cardiac APs acquisition software (Emka Technologies, France). With proper oxygenation rate, atria maintained regular AP rate for more than 2–3 h, hence allowing long recordings. Several AP parameters were assessed, including RMP, APA, AP duration at 10% (APD_10_), 50% (APD_50_), and 90% (APD_90_) of total repolarization, as well as the maximum upstroke velocity of the AP (dV/dTmax).

### 2.6 Determination of collagen abundance: Masson’s trichrome staining

Atria were fixed in 4% PFA in PBS for 48 h, washed three times with PBS and cryoprotected using a PBS-based 15% sucrose solution (4–6 h), followed by a PBS-based 30% sucrose solution (overnight). Samples were then washed using PBS, embedded in OCT blocks and rapidly frozen in liquid nitrogen. Samples were sectioned in 10 µm sections using a cryostat (Leica, Germany); tissue sections were stained with Masson’s Trichrome Stain Kit following the manufacturer’s instructions (Sigma, USA), mounted in Eukitt medium (Sigma, USA), and imaged in a Digital slide scanner (NanoZoomer S60, Hamamatsu, Japan).

### 2.7 Protein lysate and western blotting

Kir_2.1_, Kir_2.3_, and NCX-1 protein expression levels were analysed using western blot. After ketamine S/xylazine anaesthesia and euthanasia via pentobarbital i.v. injection, the heart was rapidly extracted, and the rabbit’s atria were snap frozen in liquid nitrogen and stored at −80°C. Tissue chunks were lysed using an extraction buffer containing NP40 Lysis buffer (Thermo, USA) supplemented with anti-proteases/phosphatases (cOmplete mini; PhosSTOP; Roche, USA). Total protein quantification was performed using BCA method (Pierce BCA method), following the manufacturer’s instructions; results were read using a microplate reader (Labgene, Switzerland). Samples were prepared with Laemli Buffer (Bio-Rad, USA) supplemented with β-mercaptoethanol and heated at 50–70°C for 10 min; 30 μg of proteins were loaded per lane in a 4–20% SDS-PAGE gradient Stain-Free gel (Bio-Rad, USA) for 60 min at 200 V (Mini-PROTEAN® Tetra Vertical Electrophoresis Cell, Bio-Rad, USA).

Separated proteins were transferred to a PVDF membrane using a Mini-PROTEAN® Tetra Blotting Module (Bio-Rad, USA) for 60 min at 80 V. Membranes were blocked for 1 h with 3% BSA (Bovine Serum Albumin) in 1XPBS (w/v) and incubated with primary antibodies against Kir_2.1_ (overnight, 4°C, dilution 1:400, NeuroMab, 75–210, clone N112B/14), Kir_2.3_ (overnight, 4°C, dilution 1:250, Novus, NBP3-03005), and NCX-1 (overnight, 4°C, dilution 1:1000, Invitrogen, MA3-926) diluted in antibody solution (PBS-0.1% Tween-20 + 0.3% BSA). Post-incubation, the membrane was washed five times for 10 min each with antibody solution before being incubated with HRP-conjugated secondary antibody (1 h, RT, anti-mouse for Kir_2.1_ and NCX-1, dilution 1:10000, Jackson ImmunoResearch, 115035146, 144029) (1 h, RT, anti-rabbit for Kir_2.3_, dilution 1:10000, Jackson ImmunoResearch 111035144) using the antibody solution supplemented with 0.01% SDS. After five times 10 min washes in PBS-Tween20 1%, the membrane was incubated in UltraScence Femto Plus Western Substrate (Bio-helix) and protein signal was acquired with a ChemiDoc MP Imaging System (Bio-Rad, USA). Blots were quantified using Image Lab Software (Bio-Rad, USA); K_ir2.1_ protein signal was normalized to total protein staining.

### 2.8 RNA isolation and gene expression analysis

*KCNJ2* mRNA expression levels were analysed via quantitative real-time PCR (qRT-PCR). After atrial CMs isolation, cell pellets were kept at −80°C. Total RNA was harvested using a RNeasy Mini Kit (Qiagen, Germany) according to manufacturer’s instructions and quantified using NanoDrop spectrophotometer (Quawell Q9000, Quawell Technology, USA). From total RNA, 200 ng was used to generate cDNA using the SuperScript™ VILO™ cDNA Synthesis Kit (Thermo, USA). qRT-PCR reactions were run using SensiFAST SYBR® No-ROX Kit (Labgene, Switzerland) using *GAPDH* and *KCNJ2* primers. *GAPDH* primers used were: 5′-ATGGTGAAGGTCGGAGTGAA-3′ (forward), 5′-GTAGTGGAGGTCAATGAATGG-3′ (reverse). *KCNJ2* primers used were: 5′-CCTTGGGCACAAAGGACTTA-3′ (forward) and 5′-GCTCAGTGAGGACACACACAC-3′ (reverse). qPCR was performed using MIC qPCR Cycler (Bio Molecular Systems). The thermal cycling conditions used were 95°C for 2 min, followed by 45 cycles at 95°C for 5 s, 59°C for 10 s, and 72°C for 20 s. Data were analysed using the 2-ΔCT method.

### 2.9 Pellet implantation in female rabbits

To avoid repetitive injections, DHT (150 mg) or placebo hormone-releasing pellets (Innovative Research of America, Sarasota, USA) were implanted under general anaesthesia, with ketamine/xylazine as described above, subcutaneously on the back of female animals to enable continuous chronic hormone release. As previously demonstrated, estradiol levels in young, pubertal female rabbits are relatively low, comparable to ovariectomized females and only rise during ovulation, no prior ovariectomy was performed to reduce estradiol levels.^18^ Two weeks after pellet implantation, ECGs were recorded under anaesthesia and animals were euthanized as described above to isolate atrial CMs.

### 2.10 Assessment of DHT serum levels

To quantify DHT serum levels in DHT or placebo hormone-releasing pellets implanted animals and compare them to physiological levels detected in male rabbits, blood samples were collected after euthanasia, and serum was stored at −20°C. DHT serum concentrations were analysed with a DHT ELISA kit (11-DTEHU-E01-AL, BioCat, Germany).

### 2.11 Data analysis and statistics

Data are shown as mean ± SEM for *in vivo* and *ex vivo* experiments. Statistical analyses were performed by Prism 8.0 (Graphpad, USA). *P*-values <0.05, <0.01, <0.001, and <0.0001 were considered as statistically significant and were indicated in I-V curves as *, **, ***, and **** respectively. Distribution of groups was evaluated using the Shapiro–Wilk normality test. Normally distributed groups were compared using unpaired or paired Student’s *t*-tests, where appropriate. Non-normally distributed, unpaired groups were compared using the non-parametric Mann–Whitney test. Comparisons between three or more groups were made using one or two-way ANOVA analysis of variance.

## 3. Results

### 3.1 Sex differences in atrial ECG parameters in healthy young population

**Figure 1:**
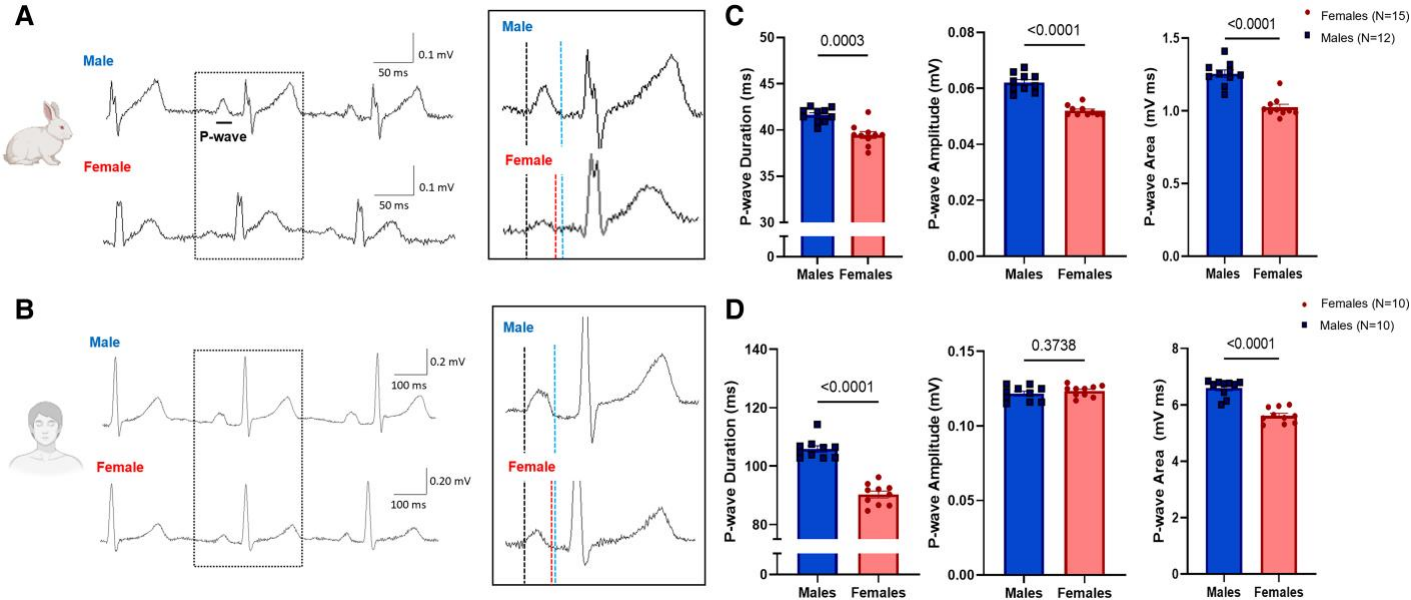
Sex differences in atrial ECG parameters in wild-type rabbits and healthy young volunteers. (*A*) Representative ECG traces from lead II recorded in surface ECGs in rabbits; top panel male animal, lower panel female animal. (*B*) Representative surface ECG traces from lead II recorded in humans, top panel men, lower panel women. Dashed lines: black represents the start of the P-wave in both sexes; red represents the end of the P-wave in female sex; blue represents the end of the P-wave in male sex. (*C*) Sex differences in P-wave parameters (duration, amplitude, and area) in rabbits. Biological replicates (*N*, animals): females = 15 and males = 12. (*D*) Sex differences in P-wave parameters in humans. Biological replicates (*N*, individuals): females = 10 and males = 10. Results are expressed as mean ± SEM. Unpaired *t*-test or Mann–Whitney test.

In 12-leads ECGs performed in healthy young rabbits (*Figure 1* and *Table 1*), males had a prolonged P-wave duration (PWD), and a larger P-wave amplitude and area in lead II, compared to healthy females (*Table 1*). Similarly, in healthy young human subjects (*Table 2*), we observed that men have a prolonged PWD and a larger P-wave area, compared to age-matched young, pre-menopausal women. Additional ECG analysis on RR interval, PR interval and heart-rate corrected QT interval (QTc) were performed (see Supplementary material online, *Figure S1*), showing the expected sex differences in QTc. No sex differences were observed in the rabbits’ atrial weight and size (see Supplementary material online, *Figure S2*). Cartoons from Biorender.com.

**Table 1:** Atrial electrocardiographic parameters in healthy rabbits

	Male rabbits	Female rabbits	*P*-value
*N*	12	15	
P-wave duration (ms)	41.61 ± 0.65	39.45 ± 0.45	0.0003
P-wave amplitude (mV)	0.062 ± 0.001	0.052 ± 0.001	<0.0001
P-wave area (mV*ms)	1.255 ± 0.026	1.024 ± 0.021	<0.0001

*N* indicates the number of biological replicates/animals. Data are expressed as average ± SEM. Unpaired *t*-test or Mann–Whitney test.

**Table 2:** Clinical characteristics and electrocardiographic parameters in healthy young women and men used to assess sex differences in atrial ECG parameters analysis

	Men	Women	*P*-value
*N*	10	10	
Weight (kg)	84.3 ± 3.7	56.7 ± 2.1	**<0**.**0001**
Height (cm)	182.7 ± 1.7	165.9 ± 2.5	**<0**.**0001**
Age (years)	31.8 ± 1.2	29.4 ± 1.7	**0**.**1060**
BMI	25.2 ± 0.9	20.6 ± 0.7	**0**.**0007**
P-wave duration (ms)	105.90 ± 1.10	90.31 ± 1.16	**<0.0001**
P-wave amplitude (mV)	0.122 ± 0.002	0.124 ± 0.001	**0**.**3738**
P-wave area (mV*ms)	6.596 ± 0.094	5.613 ± 0.085	**<0.0001**

*N* indicates the number of biological replicates/individuals. *P*-values are indicated in bold. Data are expressed as mean ± SEM. Unpaired *t*-test or Mann–Whitney test.

### 3.2 Sex differences in delayed after depolarizations in isolated atrial CMs and AP characteristics and collagen abundance in atrial tissues

**Figure 2:**
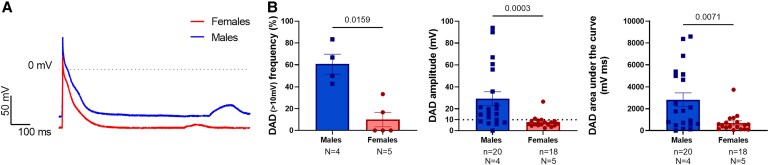
Sex DAD in atrial AP in isolated atrial rabbit CMs. (*A*) Representative AP traces in female and male atrial CMs with spontaneous DAD in control condition. (*B*) Sex differences in DAD frequency (DAD defined as delayed membrane depolarization higher than 10 mV from RMP expressed in % per animal), and DAD amplitude and DAD area under the curve. *N* indicates the number of biological replicates (*N*, animals): males = 4 and females = 5; *n* indicates the total number of individual cells recorded from males = 20, from females = 18. Results are expressed as mean ± SEM. Unpaired *t*-test or Mann–Whitney test.

During AP recordings in isolated atrial CMs, pronounced sex differences in spontaneous delayed after depolarizations (DAD) frequency and amplitude were apparent (*Figure 2*). Male atrial CMs presented significantly higher DAD frequency (males: 60.7 ± 9% vs. females: 10.0 ± 6.7%, *P* = 0.0159), amplitude (males: 29.3 ± 6.3 mV vs. females: 7.7 ± 1.2 mV, *P* = 0.0003), and area-under-the-curve (male: 2824 ± 634.5 mV.ms vs. females: 688.6 ± 202.2 mV.ms, *P* = 0.0071) compared to females (*Figure 2*).

In addition, APs were recorded in atrial rabbit tissue chunks from both sexes using the sharp-electrode technique (see Supplementary material online, *Figure S3*). In these experiments on the tissue level, no DADs were observed neither in male nor female atria. No sex differences were observed in any of the AP characteristics, particularly for the upstroke velocity, the AP amplitude or the AP duration. Importantly, regional differences in APD_10_ and APD_50_ were found only in males, with shorter APD in the LA than in the RA (see Supplementary material online, *Figure S3*).

Using Masson’s trichrome staining, we assessed collagen abundance in the atria to evaluate potential sex differences. Results showed a lower collagen abundance in female RA, as compared to male counterpart. Of note, only in females, regional differences in collagen content were observed between RA and LA (see Supplementary material online, *Figure S4*).

### 3.3 Sex differences in RMP and I_K1_ density in isolated atrial CMs

**Figure 3:**
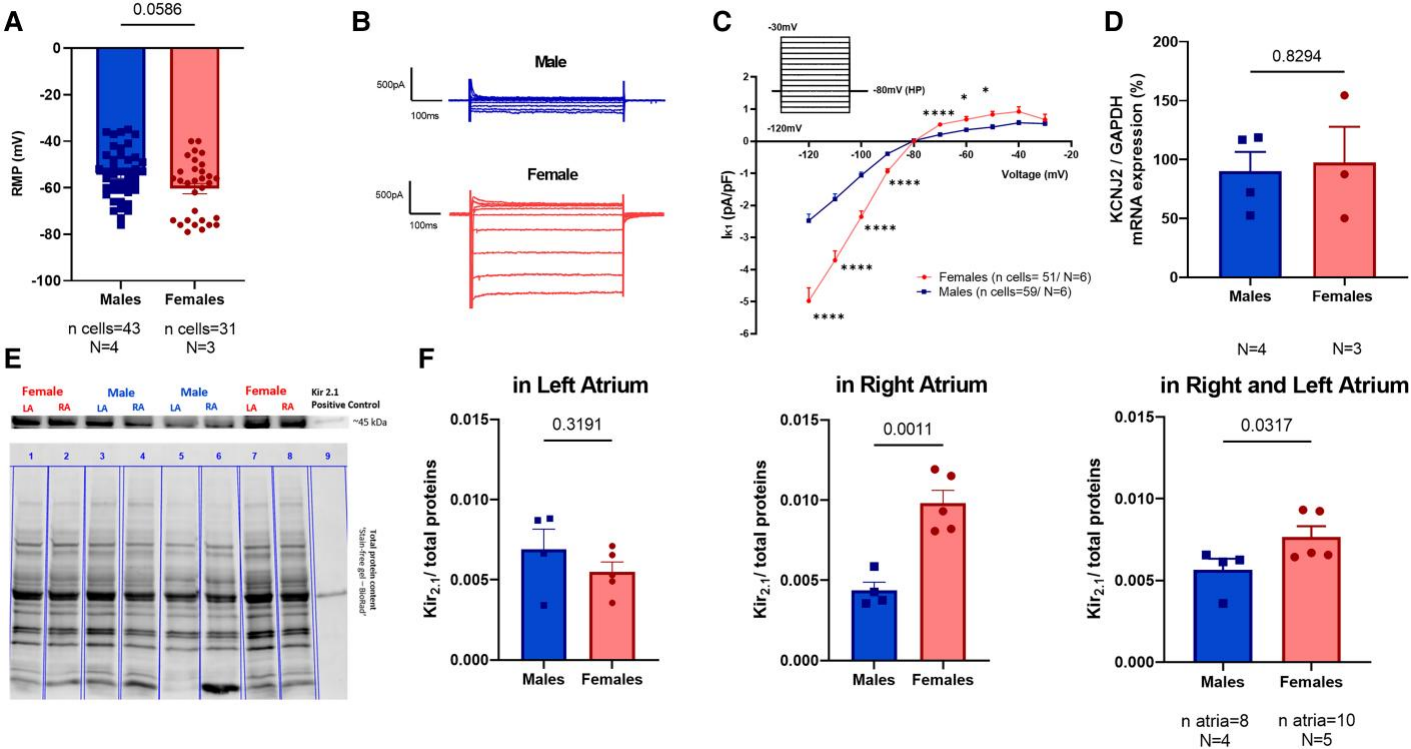
Sex differences on RMP, I_K1_ density, *KCNJ2* mRNA expression in isolated rabbit atrial CMs and Kir_2.1_ protein expression in atrial tissues. (*A*) Sex differences in RMP recorded in isolated atrial CMs using whole-cell patch-clamp in current-clamp condition. Biological replicates (*N*, animals): males = 4, females = 3, with ‘*n*’ individual cells recorded in males = 43, in females = 31. Mann–Whitney test was performed. (*B*) Representative I_K1_ traces from male and female atrial CMs recorded using protocol in (*C*). (*C*) Sex differences in I_K1_ I-V relationship in atrial CMs from both sexes. Biological replicates (*N*, animals): males = 6, females = 6, with ‘*n*’ individual cells recorded in males = 59, in females = 51. Two-way ANOVA and Sidak *post hoc* analyses were performed. (*D*) Sex differences in *KCNJ2* mRNA expression. *GAPDH* mRNA was used as housekeeping gene to normalize values. Biological replicates (*N*, animals): males = 4 and females = 3. (*E*) Representative western blot showing sex differences Kir_2.1_ protein expression (∼45 kDa) in rabbit atria. Total protein content was used to normalize protein content. (*F*) Bar graphs represent western blot quantification of Kir_2.1_ on the total protein content in the left atrium (left), in the right atrium (centre) and overall, in the atria (right). Biological replicates (*N*, animals): males = 4, females = 5, number of individual atria investigated (*n*) in males = 8, in females = 10 (two atria per rabbit). Results are expressed as mean ± SEM. Unpaired *t*-test in panel (*D*) and (*F*). *P ≤ 0.05, and ****P ≤ 0.0001.

In isolated atrial CMs, we observed sex differences in RMP. Male atrial CMs displayed a less hyperpolarized RMP compared to females (*Figure 3A*, *P* = 0.058, and Supplementary material online, *Figure S5*, *P* = 0.022). It is important to note that RMP values displayed in *Figure 3A* were obtained from cells in which I_K1_ was subsequently recorded, while the RMP values indicated in *Figure 5* stem from cells in which we recorded APs. In line with the sex differences in RMP, we found striking sex differences in I_K1_ density in atrial CMs (*Figure 3B* and *C* and Supplementary material online, *Figure S6*). At −120 mV, I_K1_ was smaller in males than in females with −2.48 ± 0.21 pA/pF in males contrasting with −4.98 ± 0.41 pA/pF in females (*P* < 0.0001). Similarly, the I_K1_ outward component measured at −60 mV was smaller in males (0.36 ± 0.05 pA/pF) than in females (0.68 ± 0.09 pA/pF, *P* = 0.0211, *Figure 3B* and *C*). As previous data from the literature reported opposite sex differences in ventricular rabbit CMs with higher I_K1_ in males,^19^ we additionally conducted I_K1_ measurements in ventricular CMs in our rabbits and could confirm these opposing sex differences in atrial vs. ventricular CMs (see Supplementary material online, *Figure S7*). At −120 mV, I_K1_ was bigger in male than in female ventricular CMs with −25.39 ± 1.80 pA/pF in males, while it was −20.59 ± 1.46 pA/pF in females (*P* < 0.0001).

### 3.4 Sex differences in *KCNJ2* expression in atrial CMs and K_ir2.1_ and Ki_r2.3_ expression in rabbit atrial tissue

qRT-PCR identified no sex differences in *KCNJ2* mRNA expression in isolated atrial CMs (*Figure 3D*). Protein analysis using western blot studies, however, showed sex differences in the K_ir2.1_ protein expression, one of the major subunits forming I_K1_ channels in the atria (*Figure 3E–G*; and Supplementary material online, *Figure S8*). Males presented a 30% lower K_ir2.1_ protein expression compared to females (protein levels in both atria (normalized to total protein content), males: 0.0076 ± 0.0007 vs. females: 0.0056 ± 0.0007, *P* < 0.05). Similarly, K_ir2.3_ protein expression tended to be reduced in male atria (*P* = 0.07) compared to female atria protein levels in both atria (normalized to total protein content), males: 0.101 ± 0.010 vs. females: 0.137 ± 0.007 (see Supplementary material online, *Figure S9*). Additionally, atrial samples were used to evaluate potential sex differences in NCX expression (see Supplementary material online, *Figure S10*). Western blot quantification revealed no differences among the two sexes in NCX protein.

### 3.5 Acute effects of sex hormones on I_K1_ density in rabbit atrial CMs

**Figure 4:**
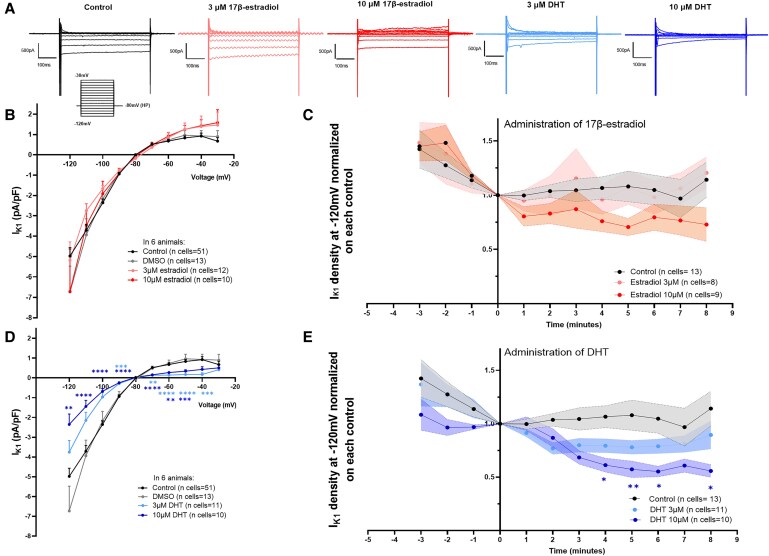
Acute sex hormones effects on I_K1_ density in female rabbit atrial CMs. (*A*) Representative I_K1_ traces of whole-cell patch-clamp recordings in atrial CMs isolated from females. (*B*, *D*) Acute effect (5 min administration) of 17β-estradiol (*B*) and DHT (*D*) on atrial I_K1_ density, in female atrial CMs from six animals (*N* = 6 biological replicates, animals). (*C*, *E*) Time course of sex hormones effects on atrial I_K1_ density in females (*C*, estradiol; *E*, DHT); plots represent current values recorded at −120 mV, normalized for the last value obtained before sex hormone administration. Results are expressed as mean ± SEM. The total number of individual cells (*n*) measured per group is indicated in parentheses. Two-way ANOVA, Dunnett in panel (*B*) and (*D*) and Sidak *post hoc* analysis in panel (*C*) and (*E*). **P ≤ 0.01, ***P ≤ 0.001, and ****P ≤ 0.0001.

We evaluated the acute effects of 17-β estradiol and DHT, first at supra-physiological concentrations (3–10 µM) on I_K1_ at physiological temperature (37°C). In female atrial CMs acutely treated with vehicle (DMSO) or 17-β estradiol, we did not detect any significant differences in I_K1_ density (*Figure 4A–C*). Female atrial CMs acutely treated with DHT, however, showed a significant I_K1_ reduction (*Figure 4A, D* and *E*). As highlighted in *Figure 4E*, after only 4 min of 10 µM DHT perfusion, I_K1_ density was reduced to about 50% of the initial current in female CMs. Similar experiments were performed using male atrial CMs. Neither 17-β estradiol nor DHT acutely modulated I_K1_ in males (see Supplementary material online, *Figure S11*). In particular, DHT reduced only the inward component of I_K1_ at very negative voltages, but no differences were observed in the outward component nor in the time course analysis (shown in Supplementary material online, *Figure S11E*), suggesting overall no meaningful effects of DHT on I_K1_ in males.

Previous studies reported that cardiac I_K1_ is modulated by protein kinase C (PKC) dependent signaling.^20^ Thus, we aimed to further examine potential underlying molecular mechanisms behind the acute DHT-induced I_K1_ reduction in female atrial CMs. We observed that DHT failed to reduce I_K1_ current in the presence of a specific PKC inhibitor (2 µM Chelerythrine), suggesting that PKC activation could be involved in the acute DHT-induced reduction of I_K1_ in female atrial CMs (see Supplementary material online, *Figure S12*).

### 3.6 Semi-chronic effects of sex hormones on I_K1_ density in rabbit atrial CMs

**Figure 5:**
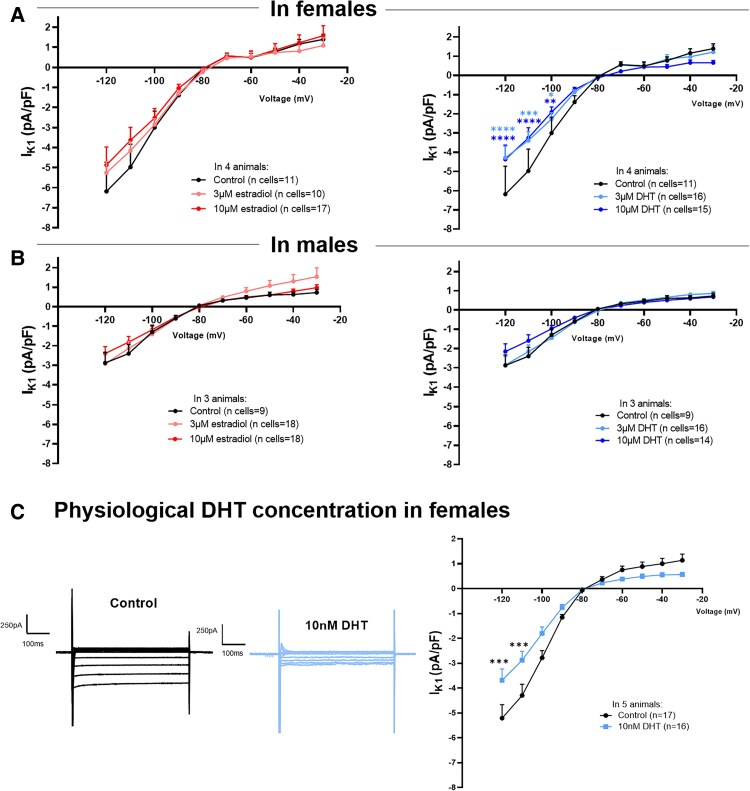
Semi-chronic (24 h) sex hormones effects on I_K1_ density in rabbit atrial CMs from both sexes and acute effect of physiological DHT concentration in female atrial CMs. (*A*, *B*) I_K1_ I-V relationship showing the semi-chronic effects of 17β-estradiol (left panels) and DHT (right panels) on I_K1_ in atrial CMs isolated from females in (*A*) (*N* = 4 biological replicates, animals) and from males in (*B*) (*N* = 3 biological replicates, animals). (*C*) (Left) Representative I_K1_ traces in a control CM and in a CM acutely (∼2 h) treated with 10 nM DHT concentration. (Right) I_K1_ I-V relationship showing the acute effects of a physiological (10 nM) concentration of DHT on I_K1_ in atrial CMs isolated from females (*N* = 5 biological replicates, animals). Results are expressed as mean ± SEM, with the number of individual cells (*n*) measured indicated in parentheses. Two-way ANOVA was performed. *P ≤ 0.05, **P ≤ 0.01, ***P ≤ 0.001, and ****P ≤ 0.0001.

To investigate semi-chronic effects of sex hormones on I_K1_, CMs isolated from both sexes were cultured for 24 h in the presence of 3–10 µM 17-β estradiol or DHT. Estradiol incubation did not affect I_K1_ density in female atrial CMs (*Figure 5A*, left) nor did it show any effect in male atrial CMs (*Figure 5B*, left). In contrast, DHT incubation strongly reduced I_K1_ density in females (*Figure 5A*, right), but had no effect in male atrial CMs (*Figure 5B*, right).

A similar set of experiments was performed to evaluate the semi-chronic DHT effect on female atrial CMs using a physiological concentration of DHT (10 nM) (*Figure 5C*). Data demonstrated that DHT significantly reduced I_K1_ density in female atrial CMs also at this much lower concentration, indicating a strong, robust effect of DHT on I_K1_ in female CM, both acutely, and chronically, and within a wide range of DHT concentrations.

### 3.7 DHT chronic effects on P-wave morphology in female rabbits

**Figure 6:**
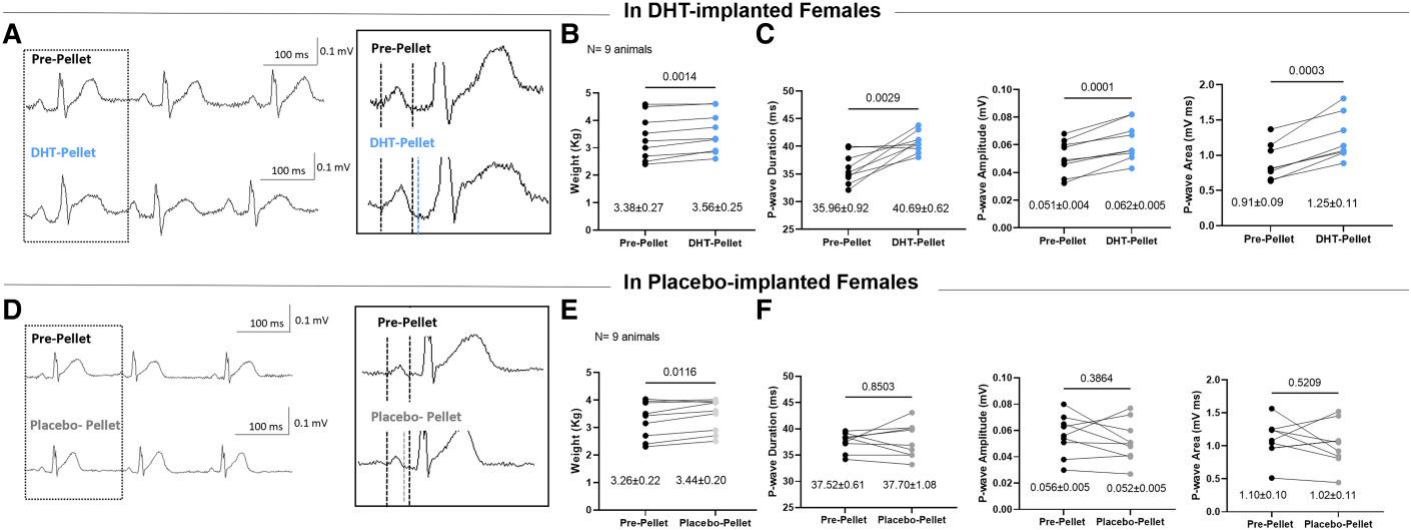
Chronic (2 weeks) DHT effects in atrial ECG parameters in rabbits. (*A*, *D*) Representative ECG traces from lead II recorded in rabbits; top panel pre-pellet implantation, lower panel same animal 2 weeks post-pellet implantation; (*A*) DHT pellet, (*D*) placebo pellet. (*B*, *E*) animals before pellet implantation, respectively. (*C*, *F*) P-wave parameters (duration, amplitude, area) in pre- and post-pellet implanted rabbits. Data represent *N* = 9 biological replicates/animals per group. Results are expressed as mean ± SEM. Paired *t*-test. (*A*, *B*, *C*) DHT pellet; (*D*, *E*, *F*) placebo pellet.

To avoid repetitive injections, DHT or placebo hormone-releasing pellets were implanted subcutaneously to enable continuous chronic hormone release. DHT levels were significantly higher in the DHT-pellet implanted females with 0.131 ± 0.019 ng/mL (∼0.45 nM) than in the placebo-pellet implanted group with 0.087 ± 0.005 ng/mL (*P* = 0.048) (see Supplementary material online, *Figure S13*). Moreover, data demonstrated that DHT levels in DHT-pellet implanted females were not statistically different from male animals (0.207 ± 0.0446, ∼0.7 nM, *P* > 0.99), suggesting that these concentrations were within the physiological range in rabbits. 12-Lead ECG were assessed in female rabbits, before and 2 weeks after DHT or placebo pellet implantation to evaluate potential DHT effects on the P-wave morphology (*Figure 6*). Data demonstrated a PWD prolongation, higher P-wave amplitude and a greater P-wave area 2 weeks after pellet implantation only in the DHT-implanted group, thus mimicking the sex differences in P-wave morphology previously observed (*Figure 1*). No significant changes were observed in the placebo-implanted group. Additional ECG analysis on RR, PR, and QTc interval were performed showing no differences between the two groups (see Supplementary material online, *Figure S14*).

### 3.8 DHT chronic effects on DAD and I_K1_ in atrial CMs

**Figure 7:**
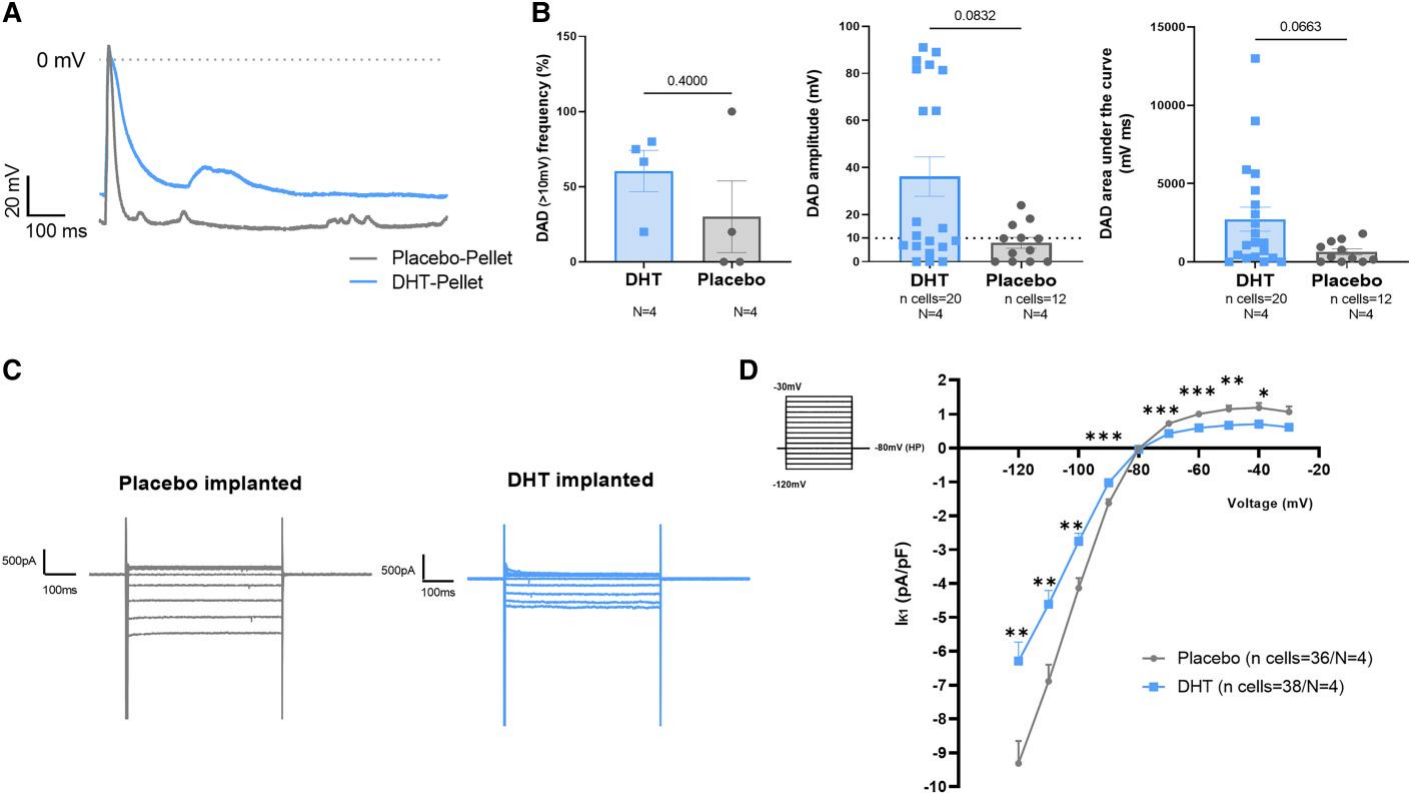
Chronic (2 weeks) DHT effects on DAD and I_K1_ density in atrial CMs isolated from pellet-implanted female rabbits. (*A*) Representative AP traces in DHT and Placebo implanted female rabbits with spontaneous DAD in control condition. (*B*) Chronic DHT effects in DAD frequency (DAD defined as delayed membrane depolarization higher than 10 mV from RMP expressed in % per animal), DAD amplitude and DAD area under the curve. Biological replicates (*N*, animals): DHT = 4, Placebo = 4; with total individual cells measured (*n*) in DHT = 20, in Placebo = 12. (*C*) Representative I_K1_ traces of whole-cell patch-clamp recordings in atrial CMs isolated from DHT and Placebo implanted female rabbits. (*D*) I_K1_ I-V relationship showing the DHT chronic effects on I_K1_ in atrial CMs isolated from implanted female rabbits. Biological replicates (*N*, animals): DHT = 4, Placebo = 4; with total individual cells measured (*n*) in DHT = 38, in Placebo = 36. Results are expressed as mean ± SEM. Mann–Whitney test in (*A*) and (*B*), Two-way ANOVA and Sidak *post hoc* analysis in (*D*). *P ≤ 0.05, **P ≤ 0.01, and ***P ≤ 0.001.

Atrial CMs were isolated from DHT- and placebo-pellet-implanted animals to investigate chronic DHT effects at the cellular level. Data obtained both in DHT- and placebo-treated atrial CMs (*Figure 7A* and *B*) show a fairly similar DAD frequency (DHT-females: 60.4 ± 13.8%; vs. placebo-females: 30.0 ± 23.8%, *P* = 0.4), but a trend towards increased amplitude (DHT-females: 36.2 ± 8.4 mV vs. placebo-females: 8.0 ± 2.3 mV, *P* = 0.08), and area-under-the-curve (DHT-females: 2727 ± 770.4 vs. placebo-females: 636.5 ± 198.4 mV.ms, *P* = 0.066) in DHT-implanted compared to placebo-implanted females.

Striking differences were found in I_K1_ density between DHT- and placebo-implanted females (*Figure 7C* and *D*). DHT-implanted females showed a smaller I_K1_ density compared to placebo-females, both in its inward and outward component. At −120 mV, I_K1_ was −6.19 ± 0.52 pA/pF in DHT-implanted rabbits vs. −9.48 ± 0.66 pA/pF in placebo-implanted rabbits (*P* = 0.002). Similarly, the I_K1_ outward component measured at −60 mV was 0.68 ± 0.06 pA/pF in DHT-implanted rabbits and 1.06 ± 0.09 pA/pF in placebo-implanted rabbits (*P* = 0.0004).

## 4. Discussion

With this study, we shed some light on sex differences and sex hormone effects on atrial electrophysiology. We specifically set out to investigate physiological sex differences in atrial electrophysiology in healthy young rabbits devoid of any pre-existing AF-induced remodelling. While previous studies,^21–23^ including work previously published by our group,^24,25^ suggest that it is nearly impossible to induce AF in young healthy rabbits devoid of any pro-arrhythmic (electrical or structural) substrate and hence to study any potential sex differences in AF inducibility in the healthy young population directly on the *in vivo* level, our cellular findings contribute to the understanding of mechanisms contributing to the higher susceptibility of young men to develop AF compared to young women, in the absence of co-morbidities. We identified pronounced sex differences in P-wave morphology, I_K1_, RMP, and delayed afterdepolarization formation, which could be mimicked with physiological DHT administration in females. These findings of a higher propensity for triggered activity in male atria likely contribute to the observed sex differences in the incidence of AF in the young population.

### 4.1 Sex differences and effect of sex hormones on P-wave morphology

We first investigated the P-wave, the ECG correlate of atrial depolarization and conduction to the AV-node.^26^ In this study, we observed that (rabbit and human) males display a prolonged PWD and an increased P-wave area compared to females. Consistent with our findings, the Copenhagen ECG study had previously observed similar sex differences in atrial ECG parameters in a bigger patient cohort: they observed that individuals with shorter PWD were younger and more likely to be women, whereas individuals with longer PWD were older and predominantly men.^26^ In this study, we provide evidence that sex differences observed in the P-wave morphology may result from sex hormones’ modulation of atrial electrophysiology—particularly by DHT—as sex differences could be mimicked by chronic DHT exposure. A PWD prolongation may suggest an overall lengthening of the time required for right and left atrial depolarization—due to differences in size or conduction velocity. In addition, computer simulation studies have proposed that a PWD prolongation may be also caused by a prolonged atrial APD.^27^ Our data acquired on rabbit atrial tissues and on isolated atrial CMs revealed no significant sex differences in atrial AP duration, and the observed sex differences in I_K1_ likely also did not contribute to the sex differences in PWD. However, other ion currents—such as the repolarizing K^+^ currents, I_Kr_, I_Ks_, and I_KACh_, and the depolarizing Na^+^ current, I_Na_, which is important for conduction velocity—may be modulated by sex hormones and potentially contribute to the observed sex differences in P-wave morphology. No sex differences were observed in size/weight of the atria. We did observe, however, that collagen abundance was smaller in female RA than in male RA, which might contribute to a slower conduction in male atria compared to females, indicating that additional sex differences in other (structural) aspects—apart from ion current characteristics—likely contribute to the observed P-wave differences in surface ECGs.

### 4.2 Sex differences in atrial ectopic triggered activity

Despite the absence of significant sex differences in AP parameters at tissue level, isolated atrial CMs exhibited sex differences in the frequency, amplitude and area under the curve of spontaneous DADs following elicited APs. Our observations of higher DAD incidence, amplitude and area under the curve in males compared to females indicate a higher propensity for triggered activity, which might be a contributing mechanism for the increased AF incidence in men in the young population. In this context, it is already well known that sex and sex hormones may affect triggered activity, regional origin of triggers, and susceptibility to pro-arrhythmic triggers.^5,28^ To further investigate potential underlying mechanisms, we analysed NCX protein expression in rabbit atria and found no sex differences. However, this does not exclude potential functional sex differences in I_NCX_ or in intra-cellular calcium handling, which could contribute to the observed differences in triggered activity.

Similarly, we also observed a trend towards a higher DAD amplitude and area under the curve in DHT-implanted females, suggesting that DHT may be a contributing factor for these sex differences in triggered activity.

### 4.3 Sex differences in RMP and I_K1_

As one mechanism underlying these sex differences in triggered activity, we identified sex differences in RMP in isolated atrial CMs with males displaying a more depolarized RMP than females. In fact, a more depolarized RMP facilitates changes in the membrane potential towards the threshold level, which could trigger APs thus contributing to the increased frequency and amplitude of spontaneous DADs.^29^

We further investigated mechanisms underlying these sex differences in RMP: we identified sex differences in I_K1_, a major contributor to the RMP in atrial CMs. Due to its conductive state around the potassium equilibrium potential and strong rectification behaviour, I_K1_ influences RMP, and phase 4 repolarization of the AP.^12^ Males exhibited a smaller I_K1_ density (reduction of around 50% at −120 mV), both in the inward as well as the outward component, compared to females. In line with these findings, we observed a 26% reduction in K_ir2.1_ protein expression in male atria compared to female, although no sex differences were identified in *KCNJ2* mRNA expression. Additionally, we identified a trend towards a lower Kir_2.3_ in males as compared to females, yet a more comprehensive protein expression analysis of all I_K1_ subunits relevant in the atria including also K_ir2.2_^14^ may further elucidate sex-specific differences in the subunit abundance within the atria.

Of note, a retrospective analysis of RA tissue, obtained during open-heart surgery in sinus rhythm and AF patients, investigated the impact of multiple clinical variables, including sex, on atrial APs.^30^ Interestingly, they observed a less negative RMP in women, associated with a lower incidence of AF. Although these patients were older and likely had comorbidities, the results are still informative and are at odds with our data in young, healthy rabbits underscoring the importance of investigating sex differences in younger populations, where sex hormones exert a more pronounced influence, and confounding factors are minimized.

### 4.4 Impact of sex hormones on atrial electrophysiology

Considering the evident sex differences in I_K1_ density, K_ir2.1_ protein expression and RMP, we explored whether sex hormones play a pivotal role in shaping these sex differences. It is well-established that sex hormones can modulate cardiac function through many cellular and molecular processes both acutely and more chronically, acting as nuclear transcription factors and/or cytoplasmic signalling activators.^31,32^ Sex hormones can activate several signalling pathways, including various protein kinases.^33–35^ Given the different mechanisms through which sex hormones can impact on cardiac electrical features, our study examined the acute, semi-chronic and chronic effects in atrial CMs. The present study elucidated that the observed sex differences in I_K1_ density could be recapitulated by DHT administration in females. Importantly, the sex differences and sex hormone effects in the potassium current I_K1_ we here revealed in the atria are *opposite to observations in the ventricles* with higher I_K1_ in male than female ventricular CMs and a testosterone-induced increase in I_K1_ observed in the same species, rabbits,^35,36^ which we could confirm in our own rabbits. These data indicate that a thorough investigation of atrial CMs/tissue is mandatory to understand the mechanisms underlying sex differences in atrial physiology and pathophysiology, to address these in targeted therapies; and that it is not sufficient to deduce potential effects from ventricular data. Notably, sex hormones possess the ability to rapidly modulate cardiac ion channels, exerting effects within seconds to minutes of their administration.^34^ Our findings suggest that DHT also exhibits a rapid, non-transcriptional modulation on I_K1_, mediated via PKC. In addition, similar DHT effects were observed semi-chronically after 24 h of incubation and chronically during hormone-pellet release, indicating that other, transcriptional effects may further contribute to the overall reduction of I_K1_ density through the modulation of K_ir2.1_ expression as also observed in male atrial CMs. In male CMs, which are constantly exposed to high DHT levels *in vivo*, additional acute or semi-chronic DHT exposure did not display any effects on I_K1_, possibly implying a target saturation effect.^37^ We assume that for similar reasons, no 17β-estradiol effects were observed in female CMs. Moreover, in DHT-pellet implanted females we observed a trend towards higher DAD amplitude and area under the curve, as compared to placebo-implanted animals, possibly due to the I_K1_ modulation by DHT. Indeed, recent studies have proven the effectiveness of an enhanced I_K1_ at suppressing DAD-mediated triggered activity.^38^ Future experiments could be conducted in orchiectomized male rabbits to finally confirm that DHT is the main driver of these observed sex differences.

### 4.5 Limitations and outlook

In this study, we primarily focused on I_K1_ and its impact on RMP and DAD formation. Here, we observed pronounced sex differences in RMP and DAD formation particularly in the isolated atrial CMs, but not at the atrial tissue level. These discrepancies likely arise from the electrical coupling of CMs via gap junctions in intact tissue, which may smoothen any potential intra-cellular variability in RMP. In contrast, isolated CMs lack this electrical coupling, making individual differences in RMP (and DAD formation) more prominently detectable. Indeed, even in isolated atrial CMs from human patients in sinus rhythm, few small DADs could occasionally be detected (albeit much less frequently than in CMs from patients in AF).^39^ Our observed sex differences on the CM level may thus indicate potential differences in the propensity to triggered activity, which may only manifest at the tissue level in case of a pro-arrhythmic (electrical or structural) substrate. By highlighting these findings, we aim to underscore the complexity of translating the cellular electrophysiological findings to intact tissue behaviour.

Notably, while atrial CMs from the LA and RA were pooled for I_K1_ current measurements as part of our initial experimental approach, the observed regional differences in Kir_2.1_ expression emerged only retrospectively, highlighting the need for future studies to systematically investigate LA vs. RA differences in I_K1_ current density.

To obtain a complete understanding of sex differences in atrial electrophysiology and atrial arrhythmogenesis as well as the observed pronounced sex differences in P-wave morphology, extensive investigation of other currents and transporters will be additionally required. This is particularly important as sex hormone effects on atrial electrophysiology are complex, often exerting opposing effects on different ion channels and calcium handling properties—similarly as observed in ventricular electrophysiology.^40,41^ Despite the scarcity of research specifically focusing on testosterone effects on atrial electrophysiology, currently available studies reported contradictory evidence regarding its beneficial or pro-arrhythmic effect. Interpreting these findings is further complicated by differences in experimental conditions, such as the use of different animal models, age groups, and sex hormone administration regimes (i.e. acute vs. chronic timing and dosage). Overall, these factors make it challenging to conclude which of the distinct testosterone-effects are prevailing ones in young healthy men. For example, pre-clinical studies in rats have shown that testosterone deficiency can pre-dispose to arrhythmias, which can be prevented by testosterone-replacement due to a stabilization of the ryanodine receptor 2,^42^ while we observed evidence of higher frequency and incidence of DAD in male rabbits’ atrial CMs, similarly as reported by Tsai *et al.*^43^ and pro-arrhythmic DAD facilitating effects of testosterone. These opposing data were, however, obtained in different species with different extent of similarities compared to human electrophysiology,^44^ making its translation to human physiology challenging. Studies in human atrial CMs showed a lower incidence of spontaneous calcium releases in aged male than in post-menopausal female CMs derived from patients with AF^45^ and opposing sex differences in RMP than in our study have been identified in atrial trabeculae from older patients undergoing cardiac surgery^30^; however, these findings reflect effects present in the older population, in which co-morbidities prevail and sex hormone levels are lower than in the young population. While the inability to induce sustained AF in healthy young WT rabbits due to the absence of a pro-arrhythmogenic substrate is a limitation in our study, our findings provide fundamental insights into sex-related electrophysiological differences already at a healthy state. This insight may guide future studies in an AF-prone model, such as the short-QT type 1 transgenic rabbit model,^24^ which is more prone to re-entry based atrial and ventricular arrhythmias due to its shortened APD/refractoriness. It will therefore be important to explore the myriad factors contributing to sex differences and sex hormone effects in atrial electrophysiology in one species and both, at young and at older age, to obtain the complete picture.

Translational perspective Herein, we investigated the role of sex and sex hormones in shaping atrial electrical features and identified pronounced sex differences in I_K1_, resting membrane potential, and delayed afterdepolarizations, which could be mimicked with dihydrotestosterone administration in females. From a clinical perspective, a better understanding of the interplay between sex, sex hormones and atrial electrophysiology provides valuable insights into possible mechanisms underlying sex-specific differences in AF incidence—particularly in the young. Overall, thorough investigations on these sex differences could facilitate patients’ risk stratification and set the stage for the development of sex-specific therapies for an improved management of atrial arrhythmias.

## 5. Conclusions

Overall, our study provides new insights into possible mechanisms underlying sex- specific differences in atrial arrhythmogenesis. Through *in vivo* and *ex vivo* experiments, we demonstrated that sex impacts on atrial electrophysiology, leading to significant sex differences in P-wave morphology, triggered activity (DAD), RMP, and I_K1_ density. In particular, our findings suggest that males present a longer P-wave and that at the cellular level, male atrial CMs exhibit higher DAD frequency and amplitude, likely due to their less hyperpolarized RMP, smaller I_K1_ density and reduced K_ir2.1_ expression levels compared to females. This increased propensity to triggered activity may contribute to the higher incidence of atrial arrhythmias observed in young men than in young women. While the observed sex differences in I_K1_ cannot explain the differences in P-wave morphology, the longer PWD in males might be partially caused by sex differences in collagen abundance in the atria, which may—in addition to the increased propensity to triggered DADs in male CMs—contribute to the increased risk for AF in males, particularly in case of further remodelling of the substrate. Furthermore, we observed that all these sex differences can be mimicked by sex hormone-treatment, particularly DHT. Hence, this study suggests that sex hormones play a role in mediating sex differences in atrial electrophysiology that might impact on sex differences in AF susceptibility in the younger population.

## Supplementary Material

cvaf074_Supplementary_Data

## Data Availability

Data generated in this study are available from the corresponding author upon reasonable request.
