# Supplementary material for: Sex and sex hormonal regulation of the atrial inward rectifier potassium current (IK1): insights into potential pro-arrhythmic mechanisms
Source: Cardiovasc Res. 2025 Apr 24;121(8):1215–27. doi: 10.1093/cvr/cvaf074 (PMC12310280; doi:10.1093/cvr/cvaf074)
Supplement: cvaf074_Supplementary_Data [file cvaf074_supplementary_data.docx]

**Supplementary Material**

**Supplemental Figure 1:**


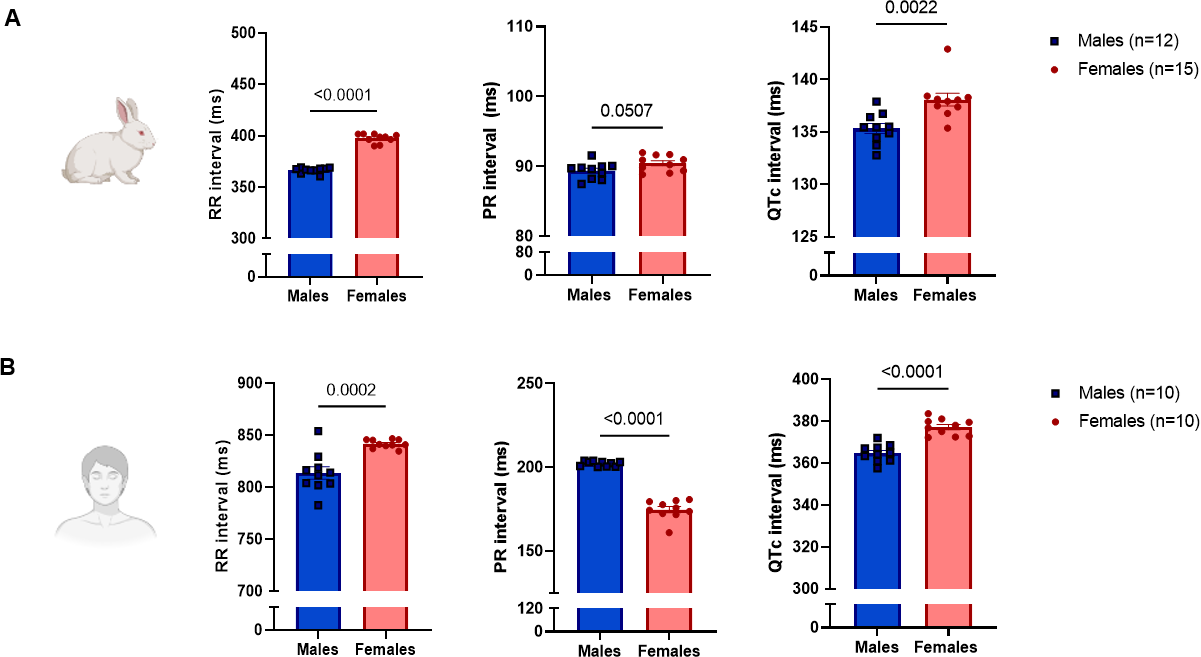


**Supplemental Figure 1: Sex differences in RR, PR and QTc interval.**

Intervals were measured in surface ECG in lead II, in wild-type rabbits (biological replicates (animals, n): Females=15, Males=12) (A) and healthy young volunteers (biological replicates (individuals, n): Females=10, Males=10) (B). Results are expressed as mean ± SEM. Unpaired t-test or Mann-Whitney test.

**Supplemental Figure 2:**

**
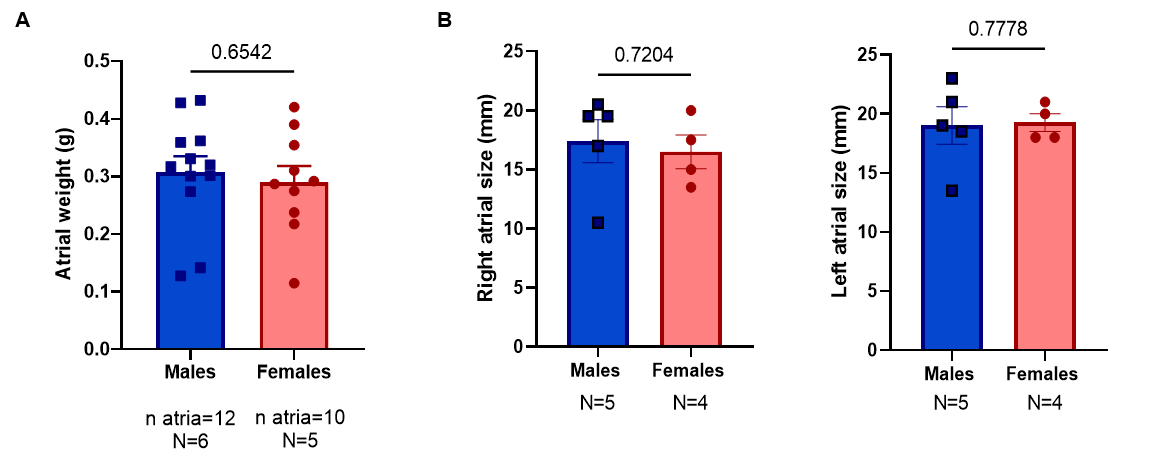
**

**Supplemental Figure 2: No sex differences in the weight (A) and size (B) of rabbits’ atria**.

N indicates the number of biological replicates (animals) and n indicates the number of individual atria investigated (two per animal in A). Results are expressed as mean ± SEM. Unpaired t-test.

**Supplemental Figure 3:**

**
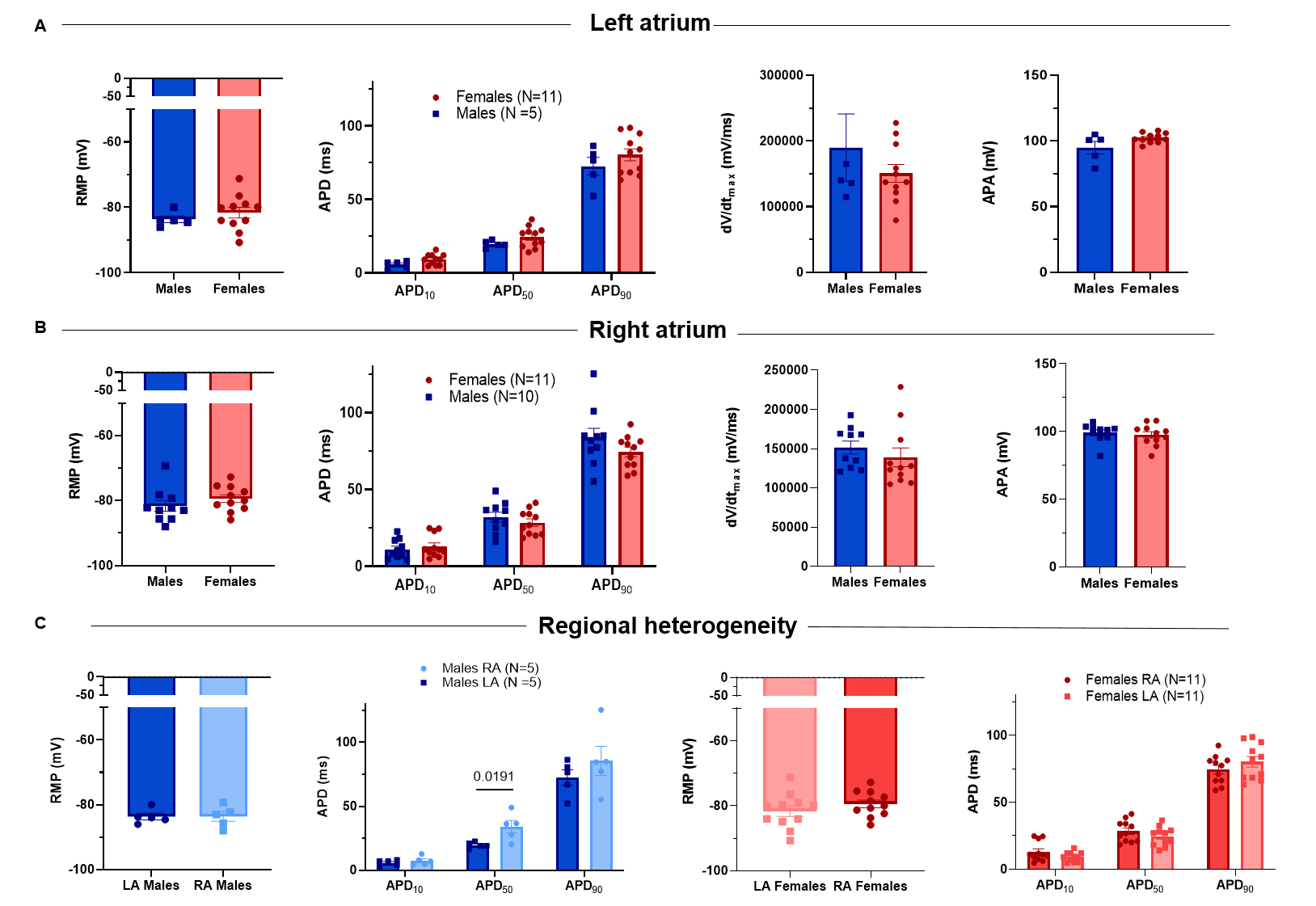
**

**Supplemental Figure 3:** **Sex differences in atrial AP parameters on tissue level.**

AP recordings from rabbit atrial tissue chunks from both sexes. Resting membrane potential (RMP), AP duration at 10% (APD10), 50% (APD50) and 90% (APD90) of total repolarization, maximum upstroke velocity of the AP (dV/dtmax) and action potential amplitude (APA) in A: left atria, in B: right atria. Statistical analysis showed no significant differences. C: Analysis of regional differences in RMP and APD in RA vs. LA in males (*left*) and females (*right*). N indicates the number of biological replicates (animals) per group. Of note, only animals for which data from both left and right atria were available are included in the analyses in C (biological replicates: N=5 males, and N=11 females). Results are expressed as mean ± SEM. Unpaired t-test.

**Supplemental Figure 4:**


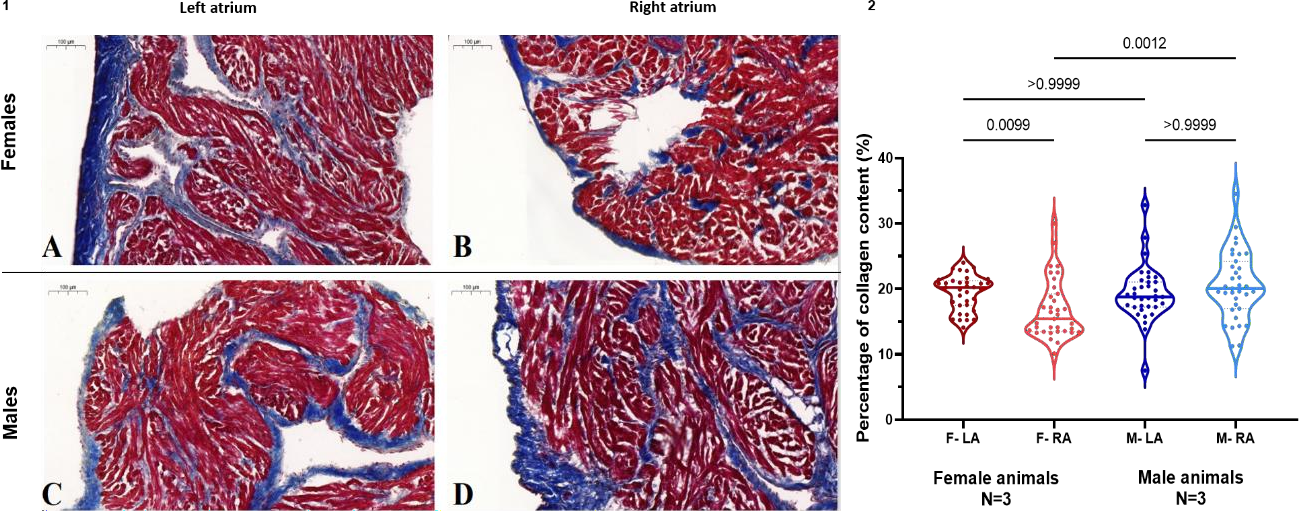


**Supplemental Figure 4: Histological assessment of collagen abundance in rabbit atrial tissue.** 1: Females A: left atrium, B: right atrium; Males C: left atrium, D: right atrium. Blue: collagen; Red: muscle; Black: nuclei. 2: Quantification of sex differences in collagen abundance in rabbit atria. N indicates the number of biological replicates (animals, Females=3, Males=3); number of total atrial regions analyzed: in Females: LA=71, RA=94; in Males: LA= 91, RA= 69. Results are expressed as mean ± SEM. One-way ANOVA.

**Supplemental Figure 5:**

**
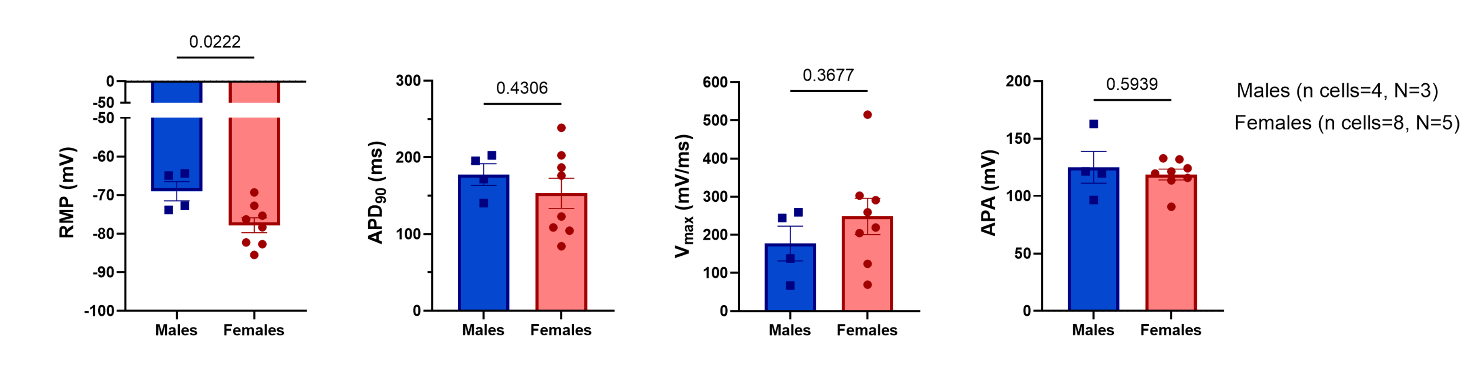
**

**Supplemental Figure 5: Sex differences in atrial AP parameters in isolated atrial CMs.**

AP parameters obtained in isolated atrial CMs from both sexes (without or only minor ≤ 30 pA injected current) in which APs were recorded. Resting membrane potential (RMP), AP duration at 90% (APD_90_) of total repolarization, maximum upstroke velocity of the AP (V_max_), and action potential amplitude (APA). Biological replicates (animals, N): Males=3, Females=5; total numbers of cells (n) from Males = 4, from Females = 4. Unpaired student t-test was performed. Results are expressed as mean ± SEM.

**Supplemental Figure 6:**


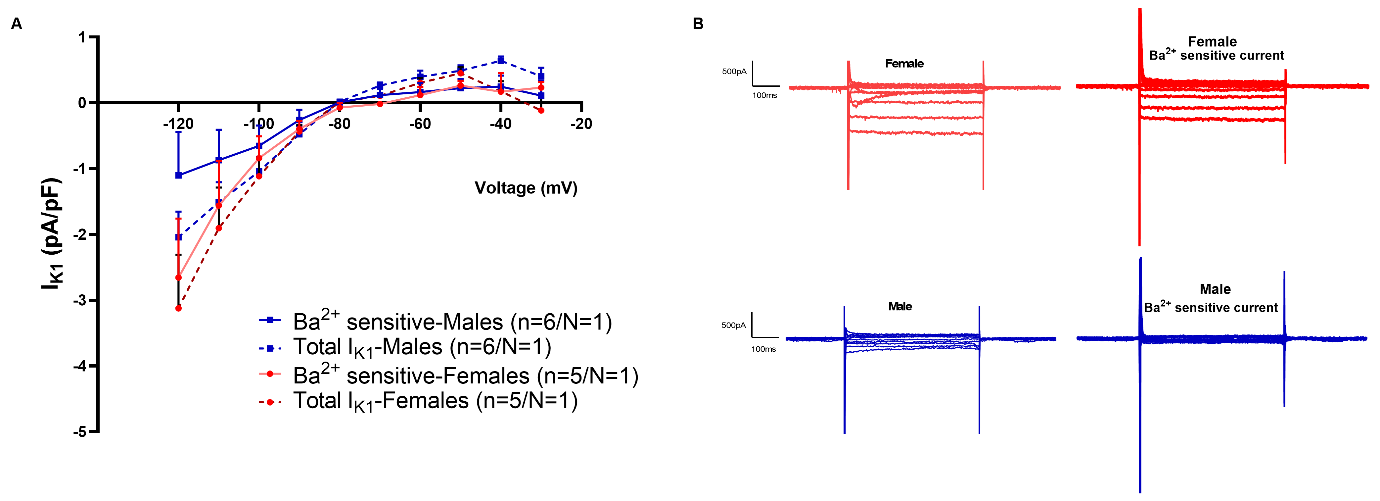


**Supplemental Figure 6: Sex differences in BaCl-sensitive I_K1_ density in atrial CMs.**A: I-V relationship of 2 mM BaCl-sensitive I_K1_ density in atrial CMs, highlighting sex differences between male and female rabbits. Data are presented as mean ± SEM, with BaCl-sensitive I_K1_ shown as solid lines and total I_K1_ as dashed lines for both sexes. Biological replicated (animals, N, Males=1, Females=1; total cells investigated (n) from Males=6, Females=5. B: Representative total (*left*) and BaCl-sensitive (*right*) IK1 traces from male and female atrial CMs.

**Supplemental Figure 7:**


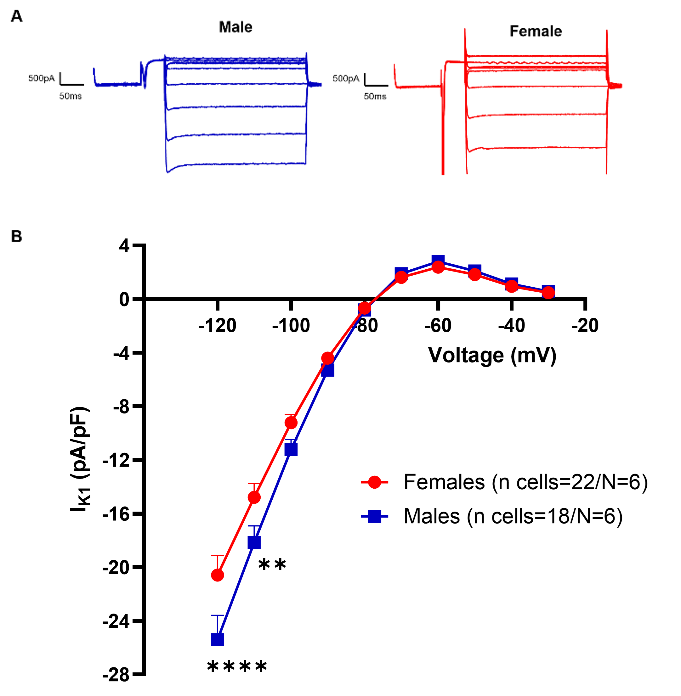


**Supplemental Figure 7: Sex differences in I_K1_ density in isolated rabbit ventricular CMs.**

A: Representative I_K1_ traces from male and female ventricular CMs using the whole-cell patch-clamp technique. B: Sex differences in I_K1_ I-V relationship in ventricular CMs from both sexes. Biological replicates (animals, N): Males=6, Females=6; total cells investigated (n) from Males=18, from Females=22. Two-way ANOVA and Sidak post hoc analyses were performed. Results are expressed as mean ± SEM.

**Supplemental Figure 8:**


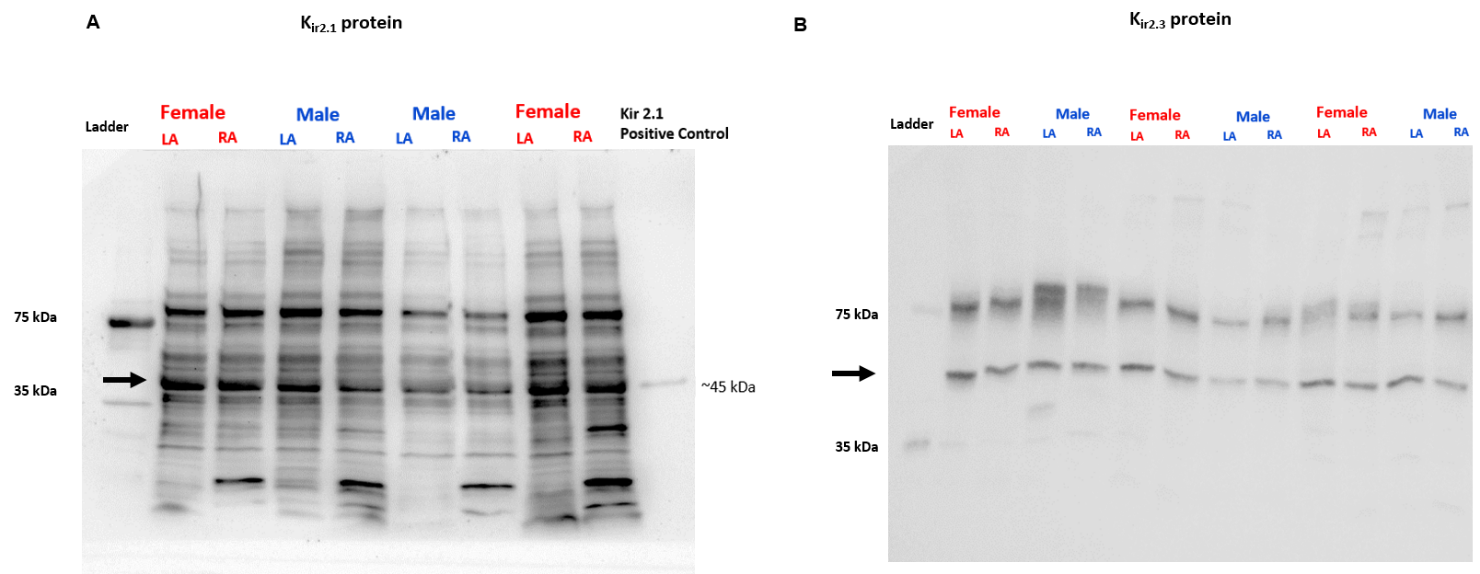


**Supplemental Figure 8:** Representative (full image) Western blots for A. Kir_2.1_ B. Kir_2.3_ protein expression in rabbit atria. Total biological replicates on the representative blots in A: N=2 males, N=2 females; in B: N3 males, N=3 females.

**Supplemental Figure 9:**


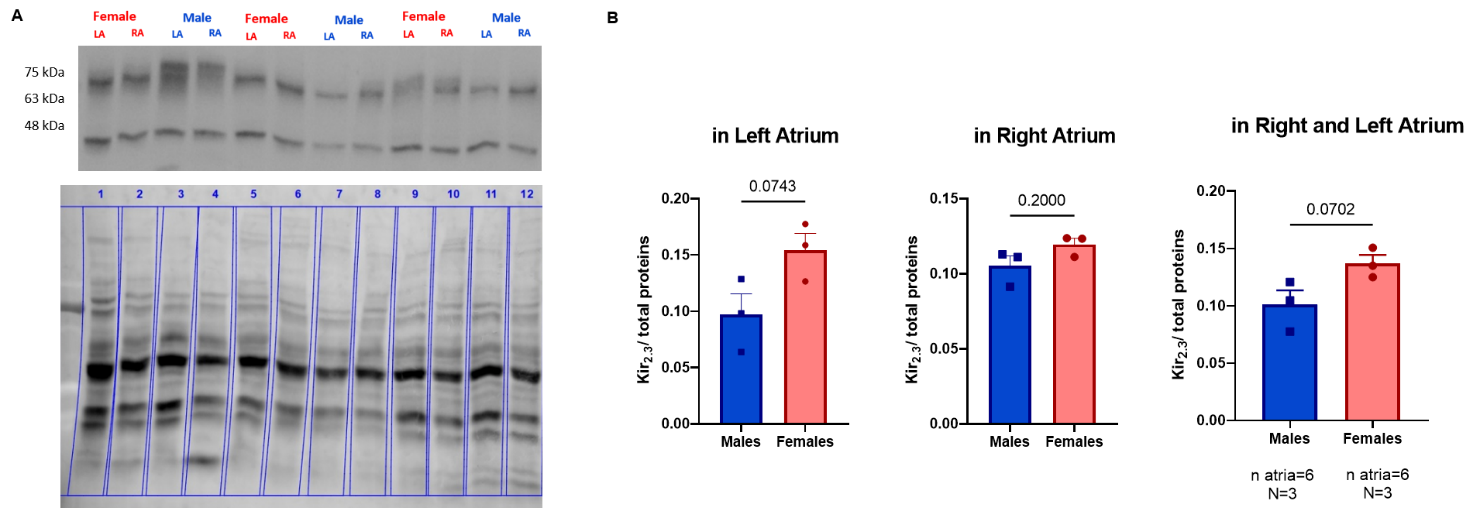


**Supplementa**l **Figure 9: Sex differences in Kir_2.3_ protein expression (~ 49kDa) in rabbit atria.**

A: Representative Western blot and (*below*) total protein content that was used to normalize protein content. B: Bar graphs represent western blot quantification of Kir_2.3_ on the total protein content in the left atrium (*left*), in the right atrium (*center*) and overall, in the atria (right). Biological replicates (animals, N): Males=3, Females=3. Number of atria (n) investigated in Males=6, Females=6 (two atria per animal). Results are expressed as mean ± SEM. Unpaired t-test.

**Supplemental Figure 10:**


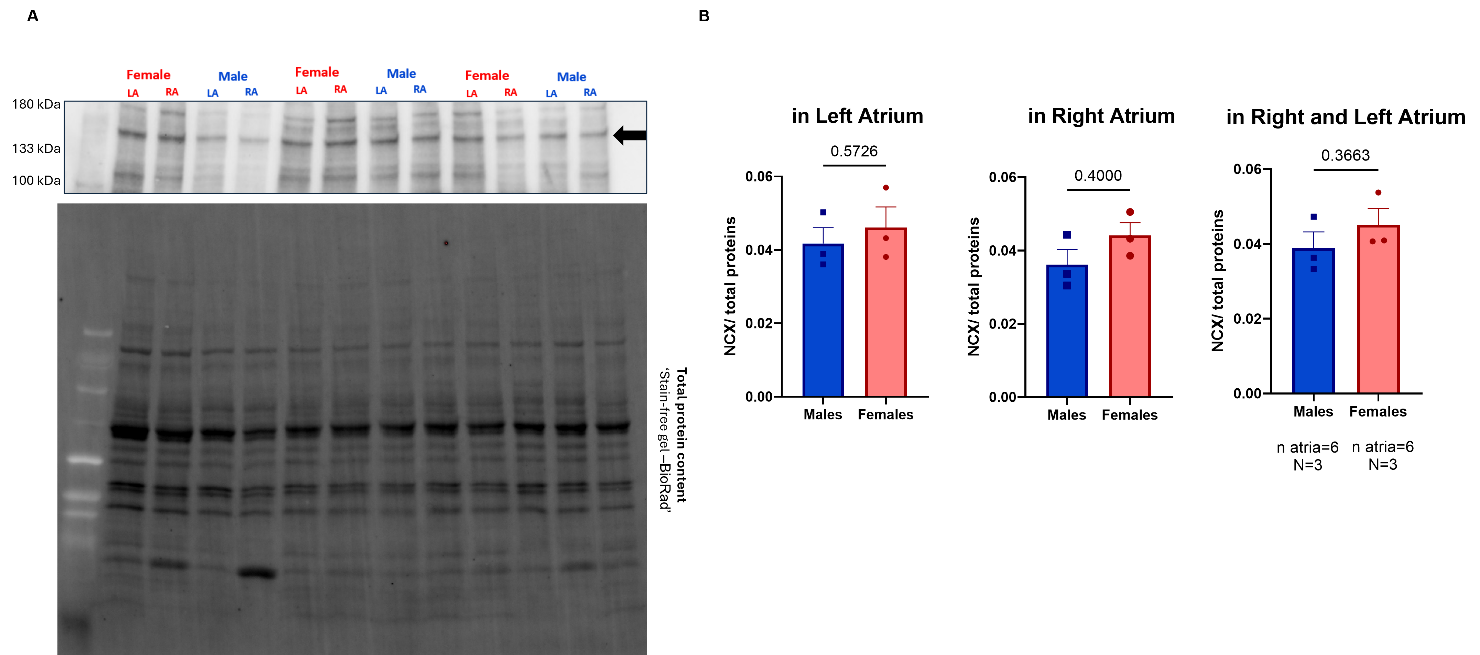


**Supplemental Figure 10: Lack of sex differences** **in NCX protein expression (~ 150kDa) in rabbit atria.** A. NCX Western blot and total protein content (*below*) which was used to normalize NCX protein expression. B: Bar graphs represent western blot quantification of NCX protein expression on total protein content in the left atrium (*left*), in the right atrium (*center*), and overall, in the atria (*right*), showed as averaged LA-RA values per animal. Biological replicates (animals, N): Males=3, Females=3; Number of atria (n) investigated in Males=6, Females=6 (two atria per animal). Results are expressed as mean ± SEM. Unpaired t-test.

**Supplemental Figure 11:**

**
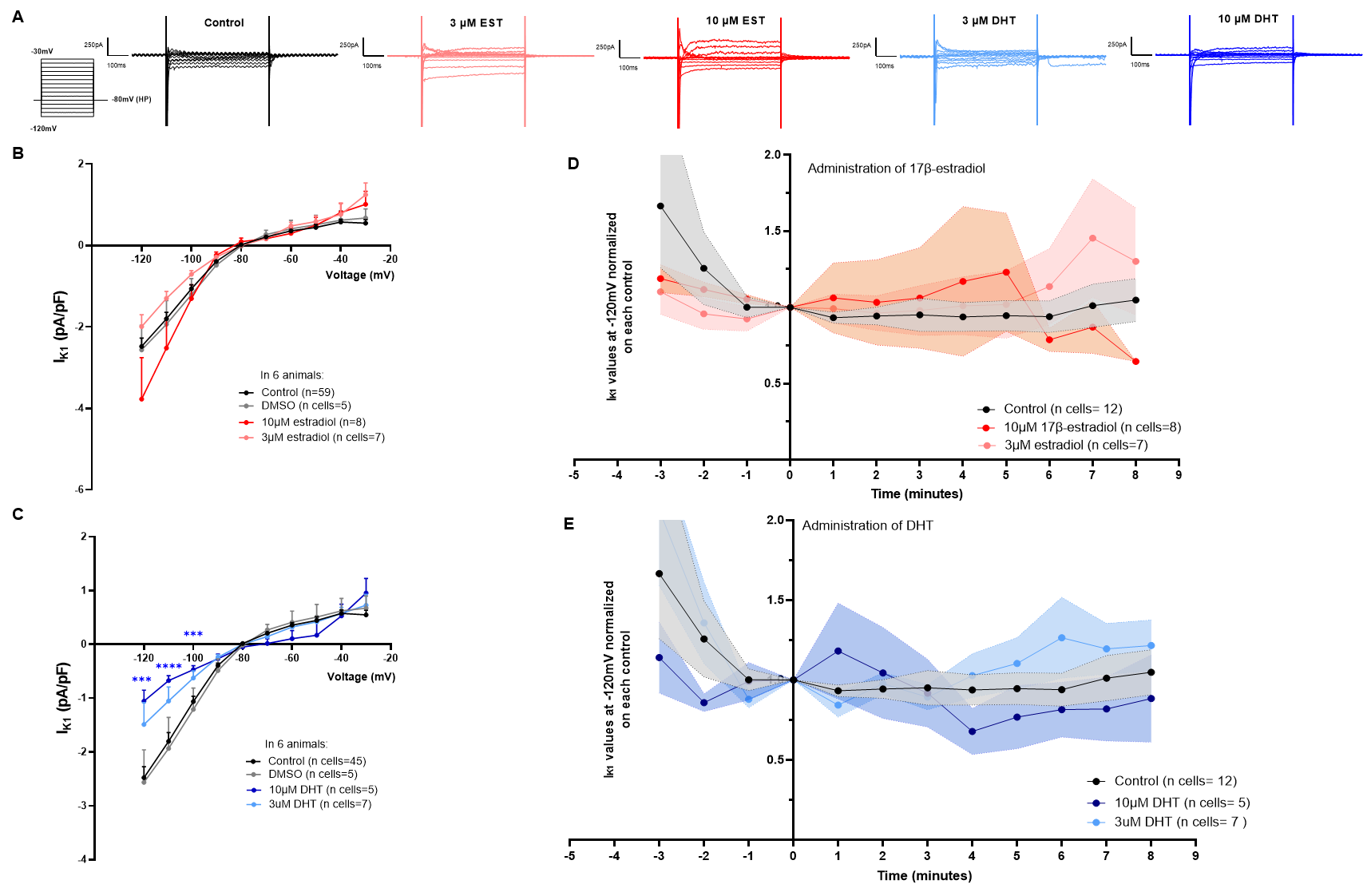
**

**Supplemental Figure 11: Acute sex hormones effects on IK1 density in male rabbit atrial CMs.** A: Representative IK1 traces of whole-cell patch clamp recordings in atrial CMs isolated from males. B, C: Acute effect (5 minutes administration) of 17β-estradiol in B and DHT in C on atrial IK1 density, in male atrial CMs isolated from 6 animals (N=6 biological replicates / animals). D, E: Time course of sex hormones effects on atrial IK1 density in females; plots represent current values recorded at –120mV. The total number of cells (n) investigated per group is indicated in parentheses. Results are expressed as mean ± SEM.

**Supplemental Figure 12:**


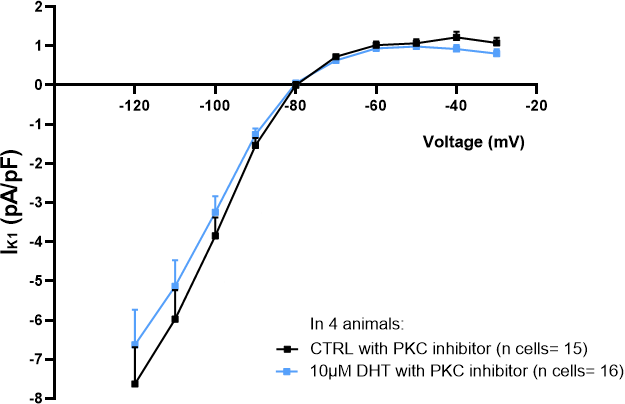


**Supplemental Figure 12: DHT acute effects on IK1 I-V relationship in atrial CMs from females with PKC inhibition.** All CMs were incubated with PKC inhibitor (alone CTRL, black; in combination with DHT, light blue). Number of biological replicates (animals, N=4), number of total cells (n) investigated is indicated in parenthesis. Two-way ANOVA and Sidak post hoc analysis was performed.

**Supplemental Figure 13:**


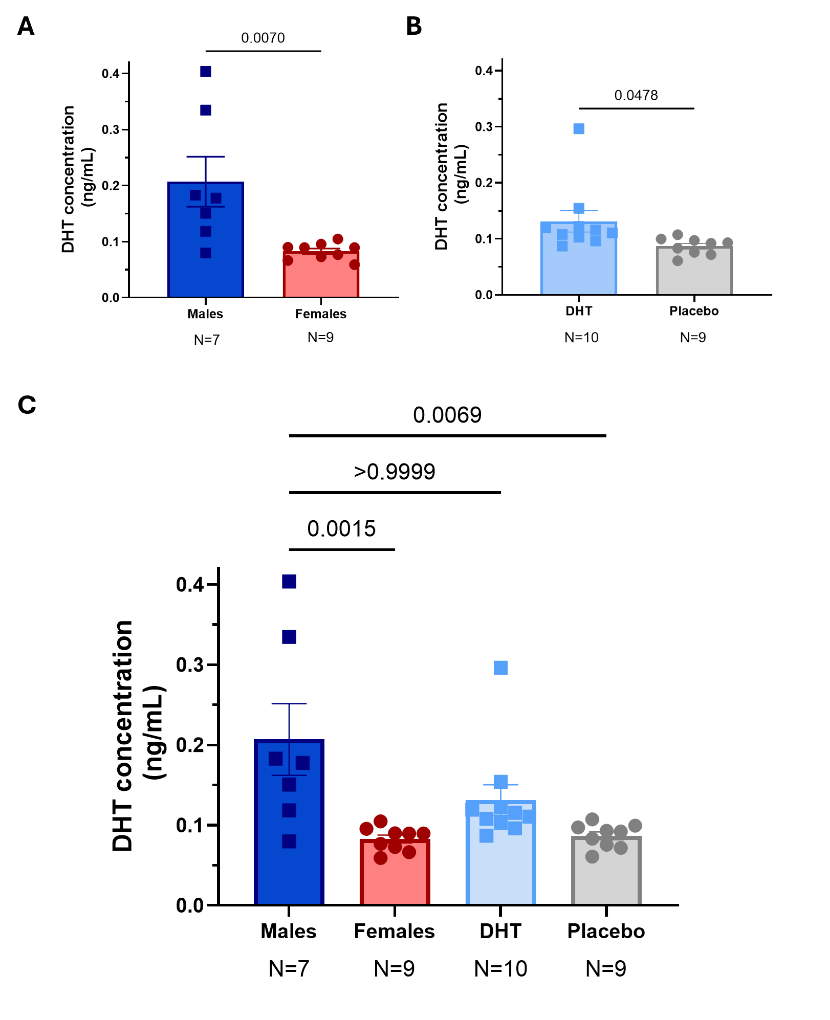


**Supplemental Figure 13: Serum DHT concentration in untreated and pellet-implanted rabbits.** Serum DHT levels were analyzed via ELISA. A: Sex differences in DHT concentrations between male and female WT rabbits used in this study. B: DHT levels obtained in DHT-implanted WT females two weeks post-pellet implantation, as compared to placebo-implanted WT females. C: Comparison of DHT levels across all groups, demonstrating that DHT-implanted WT females had significantly higher DHT levels than placebo-implanted WT females, but their levels were not statistically different from those observed in male WT rabbits. Results are expressed as mean ± SEM. N indicates the number of biological replicates (N, number of animals). Unpaired t-test or one-way ANOVA.

**Supplemental Figure 14:**


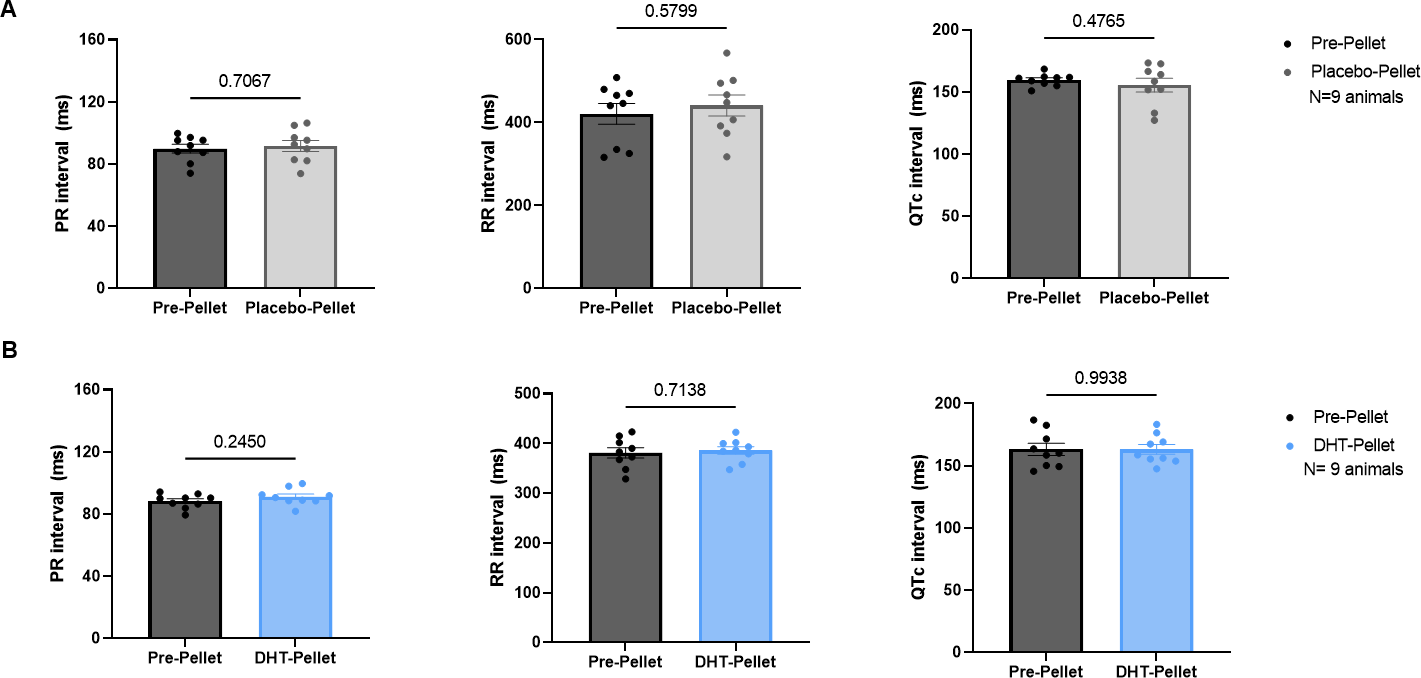


**Supplemental Figure 14: Sex differences in RR, PR, and QTc interval.**

Intervals were measured in surface ECG in lead II, in placebo (A) or DHT (B) implanted female rabbits (A). Results are expressed as mean ± SEM. N indicates the number of biological replicates (N, animals): Placebo group=9 animals, DHT group=9 animals. Unpaired t-test.
